# Spinal Control of Locomotion: Individual Neurons, Their Circuits and Functions

**DOI:** 10.3389/fphys.2018.00784

**Published:** 2018-06-25

**Authors:** Marie-Pascale Côté, Lynda M. Murray, Maria Knikou

**Affiliations:** ^1^CÔTÉ Lab, Spinal Cord Research Center, Department of Neurobiology and Anatomy, Drexel University College of Medicine, Philadelphia, PA, United States; ^2^Motor Control and NeuroRecovery Research Laboratory (Klab4Recovery), Department of Physical Therapy, College of Staten Island, City University of New York, New York, NY, United States; ^3^Graduate Center, Ph.D. Program in Biology, City University of New York, New York, NY, United States

**Keywords:** interneurons, locomotion, motoneurons, spinal neural circuits, spinal reflexes

## Abstract

Systematic research on the physiological and anatomical characteristics of spinal cord interneurons along with their functional output has evolved for more than one century. Despite significant progress in our understanding of these networks and their role in generating and modulating movement, it has remained a challenge to elucidate the properties of the locomotor rhythm across species. Neurophysiological experimental evidence indicates similarities in the function of interneurons mediating afferent information regarding muscle stretch and loading, being affected by motor axon collaterals and those mediating presynaptic inhibition in animals and humans when their function is assessed at rest. However, significantly different muscle activation profiles are observed during locomotion across species. This difference may potentially be driven by a modified distribution of muscle afferents at multiple segmental levels in humans, resulting in an altered interaction between different classes of spinal interneurons. Further, different classes of spinal interneurons are likely activated or silent to some extent simultaneously in all species. Regardless of these limitations, continuous efforts on the function of spinal interneuronal circuits during mammalian locomotion will assist in delineating the neural mechanisms underlying locomotor control, and help develop novel targeted rehabilitation strategies in cases of impaired bipedal gait in humans. These rehabilitation strategies will include activity-based therapies and targeted neuromodulation of spinal interneuronal circuits via repetitive stimulation delivered to the brain and/or spinal cord.

## Introduction

The motor cortex along with other brain areas such as the midbrain, hindbrain, cerebellum, and basal ganglia are involved in decision making and planning for movement initiation (Dubuc et al., 2008; Jordan et al., 2008; Knikou, 2012; Drew and Marigold, 2015; Grillner and Robertson, 2015). However, the quality of movement relies heavily on the translation of descending inputs and the feedback from the periphery by spinal motoneurons and interneurons that form multiple spinal neuronal networks. Interneurons are multifunctional in that their role is context-dependent, for example, a given interneuron can operate as a first-order neuron or as a last-order interneuron in different spinal reflexes, be incorporated in multiple networks, and can ultimately contribute to a variety of different movements (McCrea, 1998; Burke, 1999; Berkowitz, 2010). The complexity of the spinal interneuronal networks is also exemplified by their ability to adjust the number of motoneurons being recruited, and generate rhythmic movements that are environmentally appropriate (Sherrington, 1910; Hultborn, 2006; Kiehn, 2016).

The function of spinal interneuronal networks and their contribution to movement have been extensively investigated in animals (Jankowska, 2001, 2016a,b; Kiehn, 2016). However, little effort has been made to relate findings derived from genetic and neurophysiological studies in animals with those derived from indirect neurophysiological studies in humans (Jankowska and Hammar, 2002). Lack of recognition of similarities and/or differences between spinal interneuronal networks in animals and humans leads to a misunderstanding of neural control of movement in humans, and subsequent lack of targeted neuromodulation protocols after a neurological injury. In this review, we first discuss the direct and indirect (i.e., conditioning of monosynaptic and/or polysynaptic reflex protocols) electrophysiological methods used alone or in combination with anatomical studies to identify types, function, and integration of interneurons in animals and humans. Next, we discuss the classification system based on the expression of specific transcription factors in the developing nervous system of rodents (Goulding, 2009; Kiehn and Dougherty, 2013). Last, we discuss the evidence of spinal interneurons as controllers of locomotion, in terms of neural network functional organization, in both animals and humans.

## Classification of Spinal Interneurons

### Classification of Spinal Interneurons Based on Reflex Pathways

Historically, the anatomical and physiological properties of spinal interneurons were derived from intracellular recordings of synaptic potentials in response to afferent inputs and their interaction with other neurons. These recordings were mainly performed in the most primitive vertebrates with a limited number of neurons and simple neural networks, more specifically the lamprey (Grillner et al., 1998), tadpole (Roberts et al., 1998), and zebrafish (Fetcho et al., 2008). Our understanding of more complex networks stems from decades of studies in the adult cat spinal cord that resulted in an identification system based on the type/origin of the afferent input received, anatomical location and function (Jankowska, 2001, 2016a,b).

#### Group Ia Interneurons

Reciprocal inhibition of agonist and antagonist muscles, one of the most fundamental spinal neural pathways for neural control of movement, is largely accomblished by the IaINs. IaINs are last-order glycinergic inhibitory interneurons that mediate short-latency inhibition of antagonist motoneurons. Relaxation of an antagonist muscle following the contraction of its corresponding agonist muscle was first described by Sir Charles Sherrington in spinal cats (Sherrington, 1907; Liddell and Sherrington, 1925; Lloyd, 1946), with one interneuron being intercalated in the reciprocal inhibitory pathway and latencies compatible with monosynaptic transmission (Eccles et al., 1956; Araki et al., 1960). The synaptic actions of IaINs are exerted predominately on the somata and proximal part of the motoneuron dendrites, as revealed via recordings of postsynaptic potentials in motoneurons following spike activity of single IaINs or on the reversal potential of the Ia IPSPs in cat spinal motoneurons (Burke et al., 1971; Jankowska and Roberts, 1972).

IaINs differentiate from V1 embryonic neurons during development (Alvarez and Fyffe, 2007; Goulding, 2009), and their molecular characteristics are detailed in Section “Classification of Interneurons Based on Genetics.” IaINs are located in lamina VII, dorsal or dorsomedial to their associated motor nuclei (Jankowska and Lindström, 1972), and make multiple synaptic contacts (∼4–11 contacts) with each motoneuron (Brown and Fyffe, 1981; Rastad et al., 1990). The IPSPs amplitude and conductance values recorded from cat motoneurons following the activation of IaINs suggest that the neurotransmitter released by a single IaIN opens approximately 200 glycine-activated postsynaptic channels (Stuart and Redman, 1990), implying a powerful control of IaINs on motoneurons of antagonist muscles.

IaINs receive group I inhibition from muscles antagonistic to those that supply their monosynaptic excitation, facilitatory inputs from high threshold muscle afferents, and recurrent inhibition (for example disinhibition) from RCs and motor axon collaterals (Hultborn et al., 1971a, 1976a,b; see also Renshaw Cells). Recurrent inhibition of IaINs is of similar strength to the Ia mediated-excitation of IaINs during increasing stretch reflex activity in unaesthetized decerebrate cats (Fu et al., 1978), suggesting that recurrent inhibition controls the depth of reciprocal inhibiton. IaINs are also subject to presynaptic inhibition from flexor Ia afferents. This is supported by the significant depression of EPSPs evoked in quadriceps-coupled IaINs by quadriceps group I afferents following conditioning stimulation to the posterior biceps-semitendinosus nerve (Enríquez-Denton et al., 2000). However, in order to support this substantial neuronal interaction, anatomical labeling studies are required. In addition to the wide ipsilateral convergence and interactions, IaINs monosynaptically connect onto motoneurons and opposite IaINs of the contralateral side, suggesting a participation in the neural control of right-left limb coordination in addition to unilateral flexor-extensor coordination (Fedina et al., 1975; Hultborn et al., 1976a; Jankowska et al., 1978, 2005).

It is generally accepted that the neural organization of IaINs in humans is similar to that described in the cat, with their actions being indirectly assessed based on surface EMG recordings of the soleus H-reflex in response to common peroneal conditioning stimulation, or based on the soleus H-reflex depression following antagonistic tonic muscle activity (Crone et al., 1987; Capaday et al., 1990). The supporting evidence that IaINs mediate the soleus H-reflex inhibition upon antagonist nerve conditioning stimulation is that the (1) depression is observed at short (0–3 ms) conditioning-test intervals (Crone et al., 1987), (2) depression is present at a conditioning stimulus strength that alpha efferents are not activated (Mizuno et al., 1971), and (3) soleus-coupled RCs, at time intervals linked to motoneuron discharges, depress the reciprocal inhibition induced by activation of soleus group Ia afferents on tibialis anterior motoneurons (Baret et al., 2003). Based on these findings, we can suggest for similar synaptic actions of IaINs mediating reciprocal inhibition between antagonist ankle muscles in animals and humans.

**FIGURE 1:**
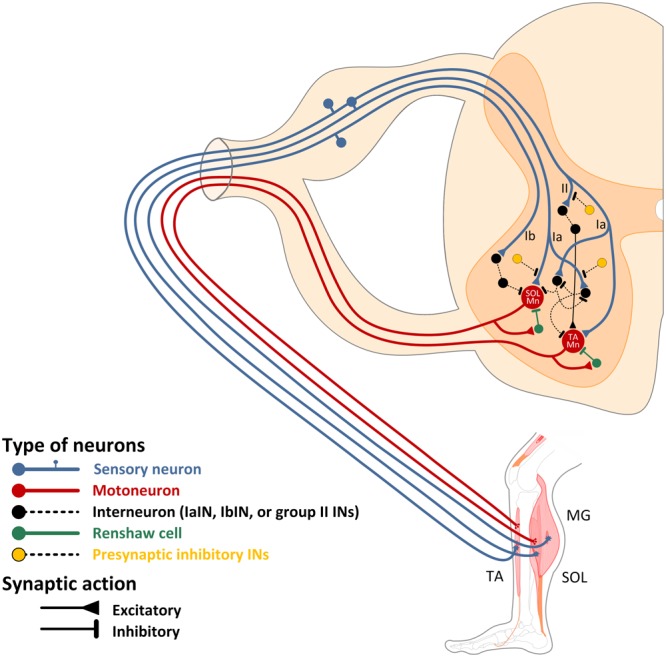
Spinal interneuronal circuits. Wiring diagram reflecting the connections of the monosynaptic Ia excitation, polysynaptic group II excitation, reciprocal Ia, RCs, and Ib inhibitory interneurons in humans. Soleus (SOL) group Ia afferents have monosynaptic excitatory projections to homonymous motoneurons and activate Ia inhibitory interneurons (IaINs) that inhibit tibialis anterior (TA) motoneurons. IaINs affected by SOL Ia afferents are inhibited by RCs activated by recurrent collaterals from SOL motor axons. Extensor-coupled IaINs inhibit contralateral flexor-coupled IaINs, and vice versa. The Ib inhibitory pathway from medial gastrocnemius (MG) to SOL motoneurons is also depicted along with presynaptic inhibitory interneurons acting on group Ia and II afferent terminals. The function of this complex spinal interneuronal circuitry is detrimental to motor output and behavior. Note that neurons within the gray matter of the spinal cord are not indicated per their laminae anatomical position due to illustration constrains.

**Figure 1** depicts the soleus and tibialis anterior Ia afferents synapsing onto tibialis anterior and soleus coupled IaINs respectively, based on evidence from studies performed in humans. Mutual inhibitory actions between “antagonistic” IaINs are also illustrated. We acknowledge the complexity of this neural circuit considering the wide range of inputs to IaINs and their widespread effect on other neurons over multiple segments (Hultborn et al., 1976b) but did not illustrate it for clarity purposes.

#### Renshaw Cells

Action of RCs on spinal motoneurons, was first described by Renshaw who also determined that the interval between the entrance of the inhibitory volley into the spinal cord and the onset of the inhibited reflex response ranged from 1.0 to 1.2 ms, suggesting that this “direct inhibition is not mediated by the direct action of dorsal root fibers on the tested motoneurons” (Renshaw, 1942, 1946). It was later shown that large motor-nerve fibers activated RCs by producing antidromic IPSPs on motoneurons with a delay of 0.5 ms following the initiation of the earliest spike in a RC (Eccles et al., 1954a). Recurrent inhibition lasts up to 50 ms with a maximal effect at approximately 10 to 20 ms in decerebrate cats (Eccles et al., 1954a). The intersegmental inhibitory action of RCs is clearly demonstrated by the action of a RC on other neighboring RCs, on IaINs, and on motoneurons located one or two spinal segments away (Ryall, 1970; Hultborn et al., 1971a,b). Intracellular recurrent IPSPs recorded from motoneurons in the isolated lumbar spinal cord of neonate rats showed that RCs are susceptible to glycinergic and GABAergic mechanisms (Schneider and Fyffe, 1992), similar to that described for the cat.

Renshaw cells are funicular and multipolar cells mostly located in lamina VII just medial to their associated motor nuclei and occasionally found in lamina IX (Jankowska and Lindström, 1971; Fyffe, 1990). Most RCs are glycinergic with only a small subpopulation being γ-aminobutyric acid (GABA)-ergic (Alvarez and Fyffe, 2007). RCs are characterized by clusters of gephyrin and glycine receptors (Alvarez et al., 1997), a feature that was recently utilized in identifying genetic markers, and determined that as IaINs, RCs also differentiate from V1 embryonic neurons (see Classification of Interneurons Based on Morphological Features; Alvarez and Fyffe, 2007; Goulding, 2009). Locally projecting motor axon collaterals release acetylcholine and glutamate activating nicotinic receptors, muscarinic, α-amino-3-hydroxy-5-methyl-4-isoxazolepropionic acid receptors, and NMDA receptors targeting RCs, that in turn affect motoneurons via a relatively simple local recurrent inhibitory circuit (Nishimaru et al., 2005; Lamotte d’Incamps and Ascher, 2008). However, many motoneurons can excite any particular RC, which in turn inhibits many motoneurons, creating a more complex convergence/divergence configuration. RCs mostly project ipsilaterally except in lower sacral segments in which some contralateral projections are evident (Jankowska et al., 1978).

The output of RCs is characterized by burst firing that outlasts the synaptic input (Eccles et al., 1954a, 1961b). RCs lengthen the inhibitory synaptic actions on motoneurons by adding a GABAergic component (Cullheim and Kellerth, 1981). However, long-lasting recurrent IPSPs are largely due to repetitive discharges of the RC. The temporal summation of successive slow IPSPs during burst firing explains the typical recurrent IPSP recorded after a ventral root volley (Eccles et al., 1954b), with IPSPs that have longer duration than those produced by IaINs. The long duration of IPSPs is substantiated by calbindin, a calcium buffering protein found abundantly in the axons and dendrites of RCs, which may facilitate summation of successive release events in a synaptic train (Blatow et al., 2003). In summary, recurrent inhibition decreases the activity of motoneurons in the subliminal fringe (Brooks and Wilson, 1959), stabilizes the discharge frequency of tonically firing motoneurons (Granit et al., 1960), inhibits motoneurons to slow contracting muscle fibers during rapid contractions (Eccles et al., 1961a), synchronizes motoneuron discharge patterns (Mattei et al., 2003), increases short-term synchronization of motoneuron discharges (Uchiyama and Windhorst, 2007), and turns off or prevents persistent inward currents (Hultborn et al., 2003). Together, these findings suggest that recurrent inhibition may have more functions in motoneuron output than once thought.

In humans, the actions of RCs in mediating recurrent inhibition has been demonstrated via indirect methods, and referred to as homonymous or heteronymous recurrent inhibition based on the type of stimulus used to condition the soleus H-reflex (**Figure 1**). The homonymous recurrent inhibition of the soleus-coupled RCs is estimated through the size of the soleus H-reflex following a supramaximal conditioning stimulus to the posterior tibial nerve at an interval of 10 ms (Bussel and Pierrot-Deseilligny, 1977). However, because this method relies on the collision of the conditioning discharge within the motor axons (orthodromic) and the motor volley from the test stimulus (antidromic) that depends on a constant after-hyperpolarization size, findings should be interpreted with caution (Hultborn et al., 2004). Heteronymous recurrent inhibition does not depend on this limiting factor, and is observed as a long-latency (up to 40 ms) soleus H-reflex depression following excitation of quadriceps Ia afferents (Bussel and Pierrot-Deseilligny, 1977; Meunier et al., 1990). The supporting evidence that recurrent inhibition mediates the soleus H-reflex depression in humans is that (1) Ib inhibition is excluded because it has a shorter latency and duration compared to recurrent inhibition, (2) after-hyperpolarization of motoneurons that prevents them from firing is excluded because the same decrease in firing probability is observed in motor units susceptible to heteronymous Ia monosynaptic facilitation, and (3) the threshold for inhibition corresponds to that of the quadriceps H-reflex discharge (Meunier et al., 1990).

In the human leg, strong heteronymous recurrent inhibition is evident between ankle extensors and knee flexors, synergistic ankle extensors, ankle extensors and hip flexors, and knee and hip flexors. Moderate to small heteronymous recurrent inhibition exists between ankle and hip flexors, and ankle and knee extensors (Meunier et al., 1990, 1994). In contrast, recurrent inhibition between knee extensors and ankle flexors is absent in cats and baboons, is strong between knee and ankle flexors in cats, and is absent from either ankle or knee flexors on distal digit motoneurons in cats (Cullheim and Kellerth, 1978; Turkin et al., 1998; Trank et al., 1999). To the authors knowledge, no data is available on the recurrent inhibition of foot and digit muscles in humans. These findings suggest that the basic principles of organization of recurrent inhibition have been preserved in animals and humans, while the observed differences are related to the connectivity pattern of Ia afferent pathways. Recurrent inhibition appears to affect motoneurons that participate in a more stereotypical activity, and is absent when skilled motor activity is needed as is the case for the digits. The reduced or absent recurrent inhibition between limb muscles tends to support that the recurrent Renshaw system is organized into inhibitory and disinhibitory projections participating in the control of groups of motoneurons based on the motor task/movement.

#### Group Ib Interneurons

The interneurons intercalated in pathways from Golgi tendon organ Ib afferents (IbINs), are characterized by the convergence of multimodal inputs including but not limited to group Ia and Ib afferents. They differentiate from embryonic neurons of various types (i.e., V0, V2, and V3) depending on whether they project ipsilaterally, contralaterally or bilaterally (see Classification of Interneurons Based on Genetics). Due to the wide convergence on common interneurons, it was proposed that this spinal neural circuit be referred to as non-reciprocal group I inhibition – excitation (Jankowska et al., 1981; Jankowska and McCrea, 1983; Powers and Binder, 1985). However, because the dominant source is the Golgi tendon organ afferents, Ib inhibition or Ib excitation is still used in the literature to describe the actions of these interneurons.

Golgi tendon organ Ib afferents mediating information regarding active and passive muscle tension are major contributors to the spinal control of movement and locomotion. Granit (1950) stated that “muscle action is aided by a central reflex mechanism of self-regulation, first speeding up, then damping its activity.” The monosynaptic reflex evoked by stimulation of the gastrocnemius nerve during contraction or stretch increased the excitability of extensor motoneurons, followed by a depression in acutely deafferented cats (Granit, 1950). It was later shown that stimulation of group I afferents produces a short-latency (1–3 ms) depression of homonymous and synergistic motoneurons through a disynaptic pathway (Laporte and Lloyd, 1952). Based on differences in threshold and conduction velocity of muscle afferents, intracellular recordings from gastrocnemius motoneurons confirmed that Ib afferent volleys in spinal cats evoke short-latency IPSPs (Eccles et al., 1957a,b). At rest, Ib afferents from extensors exert strong disynaptic inhibitory actions on extensor motoneurons, and di- or tri- synaptic excitatory actions on flexor motoneurons (Laporte and Lloyd, 1952; Eccles et al., 1957a,b).

IbINs are located in lamina VI and in the dorsal part of lamina VII, regions where group I afferents evoke the largest field potentials (Eccles et al., 1954b). Afferents from extensor muscles mainly activate IbINs that can be inhibitory or excitatory with subpopulations depressing the activity of motoneurons innervating the contracting muscle or exciting motoneurons innervating other muscles, mainly flexors. They are abundant in L6-7 spinal segments where their descending axon branches project as far as S1 in the cat (Hongo et al., 1983). IbINs receive inputs from corticospinal, rubrospinal and reticulospinal descending tracts as well as from propriospinal neurons, and group Ia/II muscle spindle afferents, cutaneous, and joint afferents. They project to alpha and gamma motoneurons, to other IbINs, and onto cells of the ventral and dorsal spinocerebellar tracts (Jankowska et al., 1983; Harrison and Jankowska, 1985). More importantly, IbINs terminals exerting IPSPs onto homonymous and synergistic motoneurons are prone to presynaptic inhibition, which gates autogenic Ib inhibition exerted onto active homonymous motoneurons (Zytnicki et al., 1990; Lafleur et al., 1992). Within a functional context, the presynaptic inhibition of transmission in Ib pathways controls the amount of contractile force at the onset of a contraction (Lafleur et al., 1992), while during rhythmic motor activity the CPG modulates the presynaptic input to IbINs (Dueñas and Rudomin, 1988; Dubuc et al., 1988). Furthermore, IbINs participate in alternative interneuronal pathways in which the fraction of interneurons selected by other IbINs through mutual inhibition or other segmental and descending inputs determines the final effect on motoneurons.

In humans at rest, Ib inhibitory actions on motoneurons is indirectly assessed by testing the effects of electrically induced synergistic group I volleys on the soleus H-reflex or the post-stimulus time histograms of single motor units. The supporting evidence that the resulting inhibition is of Ib origin is (1) the involvement of large diameter muscle afferents with the threshold of inhibition close to that of monosynaptic Ia excitation, (2) the central delay (∼ 5–6 ms) is consistent with disynaptic transmission, (3) the stimulus is below motor threshold and thus reflex depression is not due to recurrent inhibition, (4) it is widely distributed to homonymous, synergistic and antagonistic motoneurons, and (5) reflex inhibition has short duration (<10 ms) similar to that observed in cats (Araki et al., 1960; Pierrot-Deseilligny et al., 1979; Cavallari et al., 1992). However, a drawback in human experiments involves contamination of Ib inhibition by monosynaptic Ia excitation, and a decrease of Ib inhibition, via occlusion, by other interneurons being excited from the conditioning afferent volley. The weak Ib inhibition exerted from gastrocnemius medialis group I afferents onto soleus motoneurons in humans (Pierrot-Deseilligny et al., 1979) may thus be attributed to these methodological limitations, or to the possibility that Ib inhibition is stronger in the arm than in the leg as reported also in cats (Illert et al., 1976; Pierrot-Deseilligny et al., 1979). Collectively, actions of IbINs in the distal lower limb are stronger in animal compared to human, likely related to differences between bipedal and quadrupedal locomotion.

#### Group II Interneurons

Interneurons contacted by secondary muscle spindle afferents are located in the intermediate zone/ventral horn (laminae VI–VIII), and in the dorsal horn (laminae IV–V; Cavallari et al., 1987; Edgley and Jankowska, 1987; Bras et al., 1989). These two populations of interneurons differ both morphologically and functionally, with those located more ventrally having larger somata and dendritic trees than those located more dorsally, while only the intermediate neurons are considered as last-order interneurons because they synapse directly onto motoneurons (Edgley and Jankowska, 1987; Bras et al., 1989). Intermediate zone interneurons may be activated by group II muscle afferents directly or via dorsal horn interneurons (Jankowska et al., 2002).

The excitatory inputs that group II interneurons receive from the periphery are not distributed equally based on muscle origin. Interneurons located in L3-5 segments are excited by group II afferents of quadriceps, sartorius, gracilis, deep peroneal and flexor digitorum longus nerves, while group II afferents from other muscles provide input to interneurons of more caudal segments (Edgley and Jankowska, 1987; Lundberg et al., 1987). Major sources of input to the intermediate zone group II interneurons are primary and secondary muscle spindle and tendon organ afferents (Edgley and Jankowska, 1987). Low threshold cutaneous afferents and joint afferents may also excite smaller proportions of group II interneurons (Behrends et al., 1983a,b). The activity of group II interneurons is decreased by group Ib afferents via IbINs (Edgley and Jankowska, 1987), and is subject to presynaptic inhibition (Harrison and Jankowska, 1989; Rudomin and Schmidt, 1999). It was recently proposed that populations of group II and IbINs overlap, and that a given interneuron may predominantly respond to the activation of group Ib or group II afferents depending on a complex selection of inputs resulting from the functional context (Jankowska, 2016a,b).

Group II interneurons exert disynaptic excitatory and inhibitory actions on motoneurons, and tri- or polysynaptic actions on motoneurons via neurons located in the dorsal horn (Cavallari et al., 1987; Edgley and Jankowska, 1987; Bras et al., 1989), altogether evoking widespread actions to the motoneurons. One of their most significant contributions is that they coordinate the contractions of the stretched muscles, and thus their dominating action is similar to that of IbINs (i.e., excitation of flexors and inhibition of extensors; Eccles and Lundberg, 1959). However, due to their wide convergence/divergence, it is likely that group II interneurons are involved in various types of reflex circuits, including the transcortical-mediated stretch reflex (Matthews, 1991), and the short- and long-latency flexor reflex (Jankowska et al., 1967).

The number of group II afferents is similar to that of Ia afferents in the cat (Hunt, 1954), and it is possible that this is also true for humans (Pierrot-Deseilligny and Burke, 2005). The contribution of group II afferents to the neural control of movement in humans has received less attention compared to group I afferents, likely due to the lack of their selective stimulation. Excitation of group II afferents is suggested based on longer latency effects compared to monosynaptic Ia excitation, double electrical threshold from group Ia excitation, and suppressed group II actions by tizanidine (tizanidine suppresses medium latency stretch responses; Corna et al., 1995). The medium latency responses recorded from ankle muscles in response to stretch with subjects standing on a rotating platform is used extensively to delineate their function in humans (Schieppati et al., 1995; Schieppati and Nardone, 1999). This method, however, cannot be used at rest or during voluntary movement because the medium latency responses are suppressed (Schieppati and Nardone, 1999). In addition, the H-reflex modulation pattern, post-stimulus time histograms of single units, and ongoing EMG activity in response to peripheral electrical stimulation are used for the study of group II excitation. For example, medialis gastrocnemius nerve stimulation evokes a large facilitation of the semitendinosus H-reflex, and tibial nerve stimulation produces a complex excitatory pattern on the quadriceps H-reflex involving group II excitation at long interstimulus intervals (Simonetta-Moreau et al., 1999; Marque et al., 2005). The late high threshold group II excitation was confirmed from recordings in human single motor units (Simonetta-Moreau et al., 1999; Marque et al., 2005), although similar excitatory events cannot be demonstrated during movement or walking in humans. Alternatively, modulation of ongoing EMG activity following peripheral nerve stimulation, and differences in latencies of early and late excitation are reported to be the same for all three methods (Marchand-Pauvert et al., 2005). Evidence suggests that the late excitation is mediated by group II afferents in humans (Pierrot-Deseilligny and Burke, 2005), but overlap from different types of afferents mediating their action via group II or via alternative interneurons cannot be excluded. Collectively, group II interneurons have widespread effects on different classes of spinal interneurons and motoneurons that constitute one of the main barriers in parallelism of their effects in animals and humans.

#### Interneurons Involved in Presynaptic Inhibition

The synaptic efficacy of sensory feedback from the periphery entering the spinal cord is continuously filtered by presynaptic inhibition ensuring smooth movement. Karl Frank and Michelangelo Fuortes at the National Institutes of Health were the first to describe this mechanism in mammals (Frank and Fuortes, 1957). They postulated that muscle afferent volleys produce depression of the monosynaptic reflex by decreasing the EPSPs of motoneurons without concomitant changes in the time-course of EPSP depression, postsynaptic membrane potentials or excitability of the motoneurons (Frank and Fuortes, 1957). At that time, the authors theorized that this depression might be exhibited on the dendritic tree. It was later shown that the exerted inhibition coincided with DRPs and depolarization of group Ia afferent fibers in spinal cats when conditioning stimulation was delivered to the posterior biceps-semitendinosus nerve, and gastrocnemius motoneurons were activated by a maximum Ia gastrocnemius-soleus volley (Eccles et al., 1961c, 1962). In the isolated frog spinal cord, DRPs were larger from extensor muscles and smaller or absent from flexor muscles (Carpenter and Rudomin, 1973).

Presynaptic inhibition can start as early as 5 ms and can last up to 200 ms or even 1000 ms, with pulse trains evoking stronger presynaptic inhibition compared to single pulses (Eccles et al., 1961c, 1962). It is caused by PAD and involves local modulation of transmitter release at the Ia-motoneuron synapse by means of activation of GABA_A_ receptors (Rudomin, 1990; Rudomin and Schmidt, 1999). The subsequent response depends on the excitability of the cell membrane as well as chloride homeostasis that determine the direction of chloride ion displacement. GABA_A_ receptors in the afferent terminals increase the efflux of chloride ions, producing PAD and presynaptic inhibition via a reduction of the propagated action potential amplitude in the intraspinal afferent terminals (Eccles and Krnjevic, 1959; Rudomin and Schmidt, 1999). The presynaptic terminals of axo-axonal synapses in contact with the terminals of Ia afferent fibers are also thought to release GABA, which then activates GABA_A_ receptors on the afferent terminals allowing the efflux of chloride ions from the terminals and consequently their depolarization (Willis, 2006). The exclusive proprietary role of GABAergic interneurons in the process of presynaptic inhibition has been recently questioned (Hochman et al., 2010), with evidence showing an additional involvement of acetylcholine, amino acids and gap junctions in PAD genesis (Hochman et al., 2010; Bautista et al., 2012).

Presynaptic inhibition is a powerful – highly selective spinal mechanism. First, it is not distributed equally to all collaterals of the same Ia afferents thereby affecting specific groups of motoneurons differently. For example, presynaptic inhibition may be present in some collaterals, reduced in one collateral or inhibited in other collateral of the same afferent axons (Eguibar et al., 1994). Second, direct activation of last-order interneurons produce PAD in some but not in all intraspinal collaterals of the same Ia afferents, and individual afferents can display different PAD patterns (Quevedo et al., 1997; Rudomin and Schmidt, 1999; Rudomin et al., 2004). Thus, the asymmetry of presynaptic inhibition allows for selective control even though Ia and Ib afferents converge onto common interneurons. Lastly, terminal arborizations of the afferent fibers by means of presynaptic control mechanisms can function either as a simple unit or in a fractional manner, allowing information to pass only to selected groups of spinal motoneurons critical for each phase of locomotion (Rudomin, 2002, 2009).

In humans, the presence of presynaptic inhibition of Ia afferent terminals has been documented based on conditioning reflex protocols confirmed by motor unit studies, including soleus H-reflex depression following vibration of distal ankle tendons (Burke and Schiller, 1976; Morin et al., 1984), soleus H-reflex conditioning by common peroneal nerve stimulation at long conditioning-test intervals (Berardelli et al., 1987; Crone et al., 1987), and assessment of modulation of heteronymous monosynaptic Ia facilitation exerted from quadriceps Ia afferents onto soleus motoneurons (Fournier et al., 1986; Hultborn et al., 1987; Meunier et al., 1993).

Detailed investigations in animal and human provide convincing evidence that the basic organization and function of group Ia, Ib, and II interneurons including RCs and interneurons mediating presynaptic inhibiton have been preserved across species. While the methods in humans are non-direct, conditioning reflex protocols and motor unit studies have confirmed the function and organization of these spinal interneurons. Similarities include a segmental neural organization of IaINs comparable across species, wide convergence of recurrent inhibition targeted to specific groups of motoneurons based on the type of their function or the motor task/movement being executed, multidirectional synaptic actions on motoneurons by group II interneurons, and a presynaptic inhibitory network with similar neuronal characteristics. A weaker activity of IbINs between ankle synergistic motoneurons is evident in humans compared to animals, but this maybe related to differences in the distribution pattern of muscle afferents. An additional difference across species may be the depth of modulation of actions imposed by these interneurons onto motoneurons during movement, but this requires further experimentation.

### Classification of Interneurons Based on Morphological Features

Commissural and PINs were named based on their morphological features rather than on their function. They are heterogeneous, span several laminae, and are composed of multiple subpopulations based on either function or genetic markers.

#### Commissural Interneurons

**FIGURE 2:**
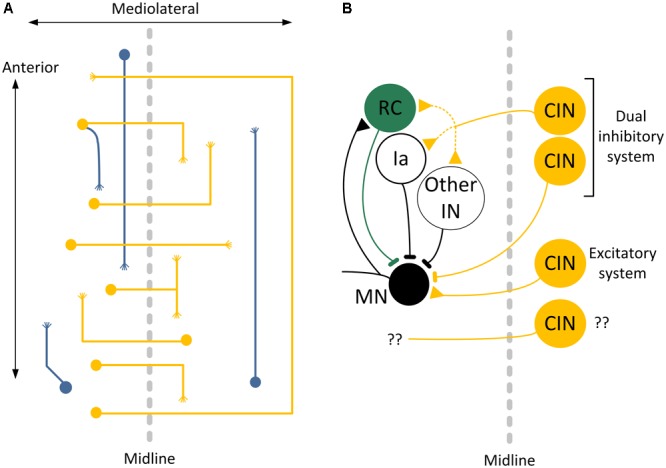
Commissural interneurons of the spinal cord. **(A)** Projections and terminations of short- and long range commissural interneurons (CINs). **(B)** CINs play an important role in the control of locomotion by projections to Renshaw cells, Ia inhibitory interneurons and other classes of inhibitory interneurons, and by direct monosynaptic excitation and inhibition to motoneurons. Adapted and modified from Quinlan and Kiehn (2007) and Chédotal (2014).

The common feature of all CINs populations is that their axons project across the midline of the spinal cord to the contralateral gray matter. In the cat and macaque monkey, CINs are located in laminae IV, V, VI, VII, VIII, and X within the cervical and lumbar segments of the spinal cord (Bannatyne et al., 2003; Jankowska et al., 2009; Soteropoulos et al., 2013). Based on their anatomical orientation and projection, CINs are divided into short-range (segmental) that project less than 1.5 segments, and long-range that project for at least 1.5 segments either rostrally (ascending) or caudally (descending) or bifurcate and project in both directions (Stokke et al., 2002; Zhong et al., 2006; see **Figure 2A**). This complex anatomical arrangement suggests the presence of multiple subpopulations, some of which still need to be functionally described.

The CINs located in the cat cervical spinal cord at C1-3 project to contralateral neck motoneurons a few segments rostral or caudal from their somata and participate in bilateral vestibulocollic reflexes, while the CINs at C3-4 project to forelimb motoneurons (C6-T1) and are active during targeted-reaching movements also providing postural stability. In rats, CINs are located in laminae VII, VIII, and X of lumbar segments (Quinlan and Kiehn, 2007), but their anatomical location in the cervical spinal cord has not yet been thoroughly established. In the neonatal rat lumbar spinal cord, the somata of CINs have large multipolar dendritic trees (Eide et al., 1999), are not of similar cell type, and each cell type has a distinct physiological function (Stokke et al., 2002). Hence, their differentiation from multiple embryonic neurons (V0, V3 and dI6; see Classification of Interneurons Based on Genetics) during development supports the non-homogenous and multifunctional nature of these interneurons.

Commissural interneurons spinal synaptic targets are motoneurons, interneurons and CINs of the contralateral side of the spinal cord. Retrograde and anterograde labeling of CINs and contralateral motoneurons of the isolated spinal cord showed that an axon bundle crossed the midline in the ventral commissure, and arborized in the contralateral ventral gray matter forming close appositions with somata and dendrites of retrogradely labeled motoneurons (Birinyi et al., 2003). Membrane potentials recorded from left and right ventral roots, while antidromic spikes identified the CINs in the presence of glutamatergic blockers, showed that CINs have excitatory glutamatergic and inhibitory glycinergic/GABAergic inputs to contralateral motoneurons (Quinlan and Kiehn, 2007). In addition, long range intersegmental CINs have excitatory and inhibitory synaptic inputs to local motoneurons (Quinlan and Kiehn, 2007; see **Figure 2B**).

Commissural interneuron receive inputs from descending motor tracts and relay somatosensory information to spinal neurons. Antidromic excitation of lumbar neurons by stimulation of the contralateral hamstring motor nuclei in the L7 segment evoked EPSPs by stimulation of group I, II afferents, low threshold cutaneous afferents, and IPSPs from the gastrocnemius-soleus or plantar nerves (Jankowska and Noga, 1990). These findings strongly support the notion that multimodal sensory inputs act on CINs in a pattern similar to the ipsilateral projecting interneurons of the same segments (Edgley and Jankowska, 1987). This phenomenon also suggests that afferents activate ipsilateral and contralateral projecting interneurons in parallel, coordinating the activity of motoneurons on both sides of the spinal cord (Edgley and Jankowska, 1987). Stimulation of reticulospinal tract fibers at the brainstem level or in the lateral funiculus of the thoracic spinal cord contralateral to the motoneurons of investigation, evoked EPSPs, IPSPs, or both in contralateral motoneurons via a disynaptic linkage (Jankowska et al., 2003). This disynaptic linkage reflects actions of CINs located in Rexed’s lamina VIII in the mid-lumbar segments (Jankowska et al., 2003).

In humans, the function of CINs is demonstrated based on crossed reflex actions from group I or cutaneous afferents on motoneurons. Specifically, crossed reflex actions are apparent based on the amplitude modulation of ipsilateral monosynaptic H-reflexes or surface EMG activity in response to stimulation of skin and peripheral nerves of the contralateral leg (Stubbs and Mrachacz-Kersting, 2009; Gervasio et al., 2013). Because crossed inhibitory responses in the soleus muscle after ipsilateral tibial nerve stimulation have 40 ms latency, the responses are likely spinally mediated (Stubbs and Mrachacz-Kersting, 2009). The contralateral soleus H-reflex depression following ipsilateral tibial nerve stimulation (Stubbs and Mrachacz-Kersting, 2009; Stubbs et al., 2011a,b), constitutes strong evidence for neural coupling between soleus motoneurons of both halves of the spinal cord. While these recordings are indirect, further research is needed on the functional activity of these spinal neural networks during locomotion in humans, and especially on their relative contribution to recovery of locomotor function after spinal cord injury.

#### Propriospinal Interneurons

The existence of neurons connecting spinal cord segments longitudinally has been acknowledged for more than a century. Sir Charles Sherrington first demonstrated that neural axons connect distal and proximal spinal segments and theorized that they communicate in order to permit long spinal reflexes (Sherrington and Laslett, 1903). These neurons were later identified as PINs to denote that they originate, project and terminate within the spinal cord. Although some PINs may have intrasegmental connections, their key role relies on forming ascending and descending connections between multiple spinal cord segments to establish relays within and across anatomical regions of the spinal cord (i.e., cervical, thoracic, lumbar). Short PINs extend their axons up to a few segments (∼1–6 segments), the number of which remains variable across the litterature. Their cell body is located in all laminae, except lamina IX, and throughout the full rostro-caudal extent of spinal cord. Short PINs form a complex network involved in a wide range of functions, including but not limited to conveying descending commands onto forelimb and hindlimb motoneurons during movement, and form relays to activate the locomotor networks. The involvement of short PINs in these functions is thoroughly described elsewhere (Alstermark et al., 1981; Alstermark and Kümmel, 1990a,b; Jankowska, 1992, 2016a,b; Zaporozhets et al., 2006; Cowley et al., 2010) and the following section will focus on long PINs involved in the spinal control of locomotion.

**FIGURE 3:**
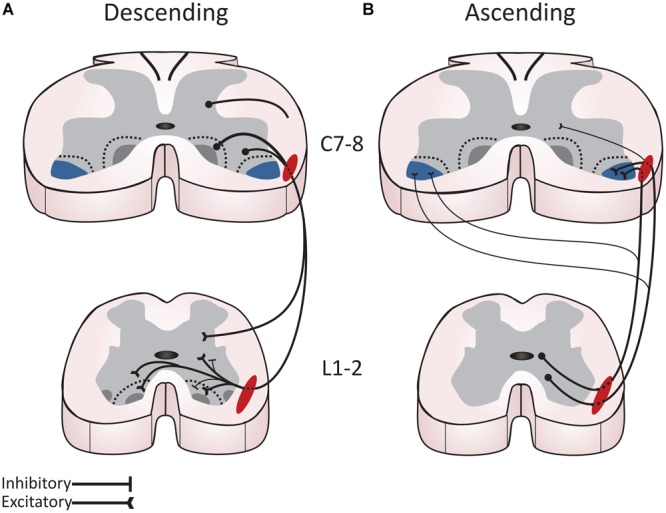
Long propriospinal interneurons (PINs) reciprocally connect the cervical and lumbar spinal cord and contribute to locomotor movement in rodents. **(A)** Descending PINs form a complex bilateral system with excitatory and inhibitory components to mediate interlimb coordination and to relate information to the CPG. Their cell body is located through all laminae of the cervical cord, but most originate from laminae VII-VIII and the deep dorsal horn. They project to non-motoneuronal elements in similar proportion to the ipsilateral and contralateral rostral lumbar cord through the ventrolateral funiculus (red). The ipsilateral population terminals are evenly distributed throughout the gray matter, whereas the projections of the contralateral population are concentrated in laminae VII-VIII. The vast majority of descending PINs are excitatory both on the ipsilateral or contralateral side but the small inhibitory population terminates ipsilaterally. **(B)** Ascending PINs form a powerful ipsilateral excitatory pathway from the rostral lumbar cord to motoneurons controlling proximal muscles of the forelimbs. Ascending PINs originate mostly from the intermediate gray in the lumbar spinal cord and preferentially project ipsilaterally with a very limited number of terminals found contralaterally. They project to the intermediate gray matter and the ventral horn throughout the length of the cervical spinal cord. However, a large proportion directly connects to motoneurons in ventrolateral motor nuclei (blue) in caudal cervical segments controlling muscles of the elbow and shoulder. The thickness of the lines represents more PINS. Figure adapted and modified from Flynn et al. (2011) and Brockett et al. (2013).

A substantial population of long ascending and descending PINs connects the cervical and lumbar enlargements via the ventrolateral funiculus. Long descending PINs with cell bodies located in the cervical enlargement terminate bilaterally in all lumbar segments of the spinal cord with a small subpopulation (∼12%) terminating close to midline (see **Figure 3A**; Giovanelli Barilari and Kuypers, 1969; Reed et al., 2006; Brockett et al., 2013; Flynn et al., 2017). Although there is an approximately even number of long descending PINs extending their axon ipsilaterally and contralaterally, they exhibit a widely different projection pattern. Ipsilateral PINs are diffusely distributed across all laminae of the gray matter but more prevalent in the deep dorsal horn (lamina IV–VI), intermediate gray (VII–VIII) and ventral horn while contralateral PINs are concentrated at the ventromedial border of laminae VII–VIII (Reed et al., 2006, 2009; Brockett et al., 2013; Flynn et al., 2017). Long descending PINs are mostly excitatory whether projecting ipsilaterally or contralaterally. Interestingly, the remaining inhibitory population is mostly projecting ipsilaterally (Flynn et al., 2017), but its specific role remains to be determined. The highest concentration of terminals in the lumbar spinal cord of rodents is found in L1–L2, and segments that are suggested to contain the dominant elements of the rhythmogenic networks (Cazalets et al., 1995; Kjaerulff and Kiehn, 1996; Cowley and Schmidt, 1997; Ballion et al., 2001; Brockett et al., 2013).

Ascending projections originating from the lumbar spinal cord terminate both ipsilaterally and contralaterally in the cervical spinal cord (see **Figure 3B**, Giovanelli Barilari and Kuypers, 1969; Matsushita and Ueyama, 1973; Dutton et al., 2006; Reed et al., 2009). Lumbar ascending PINs predominantly form ipsilateral projections that terminate in the intermediate gray matter and ventral horn throughout the length of the cervical spinal cord. Further evidence suggests that a large proportion of these projections terminate in the ipsilateral ventrolateral motor nuclei of C7-8 segments with contralateral projections to the contralateral nucleus being sparse (Brockett et al., 2013). Electrophysiological experiments and immunostaining suggest that an important proportion of long ascending PINs make direct contact with motoneurons whether they are ipsilateral or contralateral with the vast majority being excitatory (Miller et al., 1973; Brockett et al., 2013). The prominence of ascending PINs originating from the lumbar intermediate gray matter terminating ipsilaterally suggests a direct control of motoneurons, and premotor neurons, in the most caudal cervical segments controlling elbow and shoulder muscles (Sterling and Kuypers, 1968; Giovanelli Barilari and Kuypers, 1969; English et al., 1985; Alstermark et al., 1987; McKenna et al., 2000; Ballion et al., 2001; Reed et al., 2009). Together, these studies suggest that long PINs preferentially connect the caudal cervical and the rostral lumbar segments of the spinal cord (Miller et al., 1973; English et al., 1985; Reed et al., 2006, 2009). The anatomical and functional arrangement of this propriospinal network strongly suggests a role in synchronizing the lumbar and cervical CPGs and mediating interlimb coordination during locomotor movement. Long PINs were also shown to synchronize forelimb and hindlimb activity during stepping (Miller et al., 1973; Jankowska et al., 1974). Inhibition of long ascending PINs can disrupt interlimb coordination (Pocratsky et al., 2017), and blocking transmission in the thoracic cord uncouples the rhythmic activity from the cervical and lumbar enlargements (Ballion et al., 2001; Juvin et al., 2005).

Similar mechanisms persist to coordinate movement of arms and legs in humans in which PINs also contribute significantly to neural coupling between cervical and lumbar spinal segments. Activation of PINs is evident from changes of motoneurons excitation mediated by peripheral volleys or cutaneous-mediated depression of the descending motor command passing through the propriospinal relay. For example, the non-monosynaptic facilitation of the quadriceps H-reflex and peaks of excitation of quadriceps motor units following common peroneal nerve stimulation (Forget et al., 1989a,b; Simonetta-Moreau et al., 1999), is attributed to the activation of short lumbar PINs by group I afferents. The changes in the central delay of lumbar PINs in a rostro-caudal order (Chaix et al., 1997) suggests that this population is located rostral to motoneurons, as is the case for the cat. Static contralateral arm flexion decreases the ipsilateral soleus tendon reflex, whereas static contralateral arm extension produces reflex facilitation (Delwaide et al., 1977). The exact opposite reflex modulation pattern is observed when the contralateral limb is flexed or extended (Delwaide et al., 1977). Similarly, ipsilateral and contralateral sinusoidal active arm movements depress soleus H-reflex excitability in seated and standing human subjects (Knikou, 2007). These findings support the neural coupling between arms and legs during movement. As reflex responses in the upper or lower limb muscles have also been postulated following stimulation of sensory nerves in the wrist or ankle at rest (Zehr et al., 2001), movement mediated activity of spinal neural circuits can readily be excluded. More importantly, the lumbosacral cord potentials with an onset latency of 12 ms and excitation threshold at or above that of motor fibers, supports the existence of a fast-conducting propriospinal pathway in humans (Sarica and Ertekin, 1985).

Propriospinal interneurons in humans may also play an important role in motor recovery after spinal cord injury as PINs were identified in animals as key players to form relays or detours in the injured spinal cord and thus are critical in promoting functional recovery (Bareyre et al., 2004; Vavrek et al., 2006; Courtine et al., 2008; Flynn et al., 2011; Benthall et al., 2017) by being more resistant to injury and degeneration (Conta and Stelzner, 2004). The plastic potential of PINs and contribution to recovery in humans is supported by evidence of reemergence in interlimb reflexes 6 or more months after a cervical spinal cord injury (Calancie et al., 2005). CINs and PINs connecting the two halves of the spinal cord and cervicothoracic and thoracolumbar spinal segments have a similar basic organization across species. Their importance in human locomotion has received less attention, but spinal hemisection experiments in animals (Martinez et al., 2012) provides strong evidence supporting their significant contribution to locomotion. The neuronal pathways connecting the two halves of the spinal cords have not been examined in great detail during locomotion in humans after spinal cord injury, and warrants further research.

### Classification of Interneurons Based on Genetics

**FIGURE 4:**
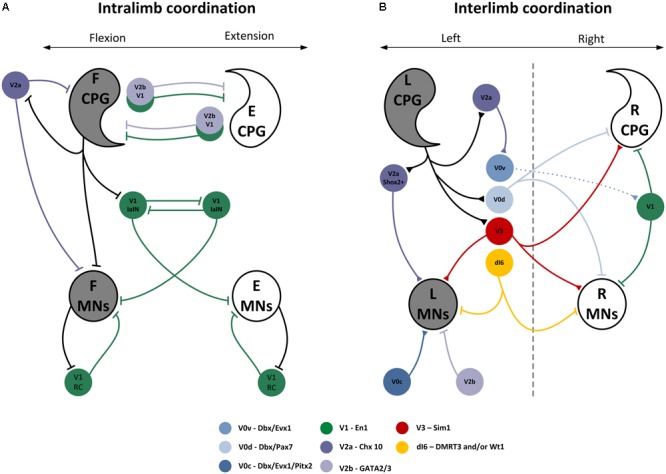
Genetically identified interneurons contributing to locomotion. Schematic of the synaptic connectivity of genetically identified populations of interneurons developing from the ventral spinal cord and involved in **(A)** intralimb and **(B)** interlimb coordination during locomotion. Experimentally demonstrated projections are illustrated by a solid line and predicted connectivity with a dashed line. Figure was developed based on Kiehn (2011, 2016) and Gosgnach et al. (2017).

The advancement of molecular genetic techniques in the last two decades has extended new possibilities to identify neurons derived from genetically well-defined embryonic cells that are expressing specific transcription factors. Combining molecular genetics with classical electrophysiological techniques accelerated our understanding of the generation and modulation of the locomotor pattern. Spinal interneurons are divided into fundamental populations expressing specific transcription factors originating from distinctive developmental progenitor domains on the dorso-ventral axis. These include six classes with dorsal origin (dI1-dI6), and four classes with ventral origin (V0, V1, V2, and V3), which are considered as putative constituents of the spinal locomotor neural networks. Exhaustive reviews of the organization of interneuronal spinal circuits and their involvement in locomotion can be found elsewhere (Jessell, 2000; Goulding, 2009; Arber, 2012; Kiehn, 2016; Gosgnach et al., 2017). This section emphasizes the diversity of spinal interneurons located ventrally or dorsally (V0, V1, V2, V3, and dI6) closely associated to the locomotor CPG, their extensive pattern of connectivity and their involvement in generating flexible and adaptive locomotor output (see **Figure 4** and Evidence From Animal Studies Using Molecular Genetic Approaches and Classical Electrophysiological Methods).

The V0 interneurons derive from Dbx-1 expressing progenitor cells and are mainly composed of CINs projecting axons to the contralateral side of the spinal cord. They can be further subdivided into a ventral subpopulation (V0v) composed of excitatory interneurons that express the transcription factor Evx1/2, and a dorsal subpopulation (V0_D_) that are inhibitory and do not express Evx1/2. Together, V0v (25%) and V0_D_ (70%) account for the majority of V0 population but other smaller subpopulations have been described including the cholinergic V0c subpopulation which, as V0v, is Evx1/2^+^ but synapse onto ipsilateral motoneurons (Zagoraiou et al., 2009). Through their monosynaptic contact to contralateral motoneurons, V0_D_ contributes to the right-left coordination at slow speeds, whereas V0_V_ are recruited as the speed of movement increases through the activation of a last-order motoneuron located in close vicinity to the target motoneuron on the contralateral side (Lanuza et al., 2004; Crone et al., 2009; Talpalar et al., 2013).

In contrast to V0 and V3, V1 and V2 classes of interneurons project almost exclusively ipsilaterally. V1 interneurons are inhibitory interneurons expressing the transcription factor Engrailed-1. Thirty percent will differentiate into RCs and IaINs with synaptic contacts onto motoneurons and other IaINs (Wenner et al., 2000; Alvarez et al., 2005) but most of the population remains to be characterized in term of synaptic target, molecular signature and functional role. Studies on the neural properties of these interneurons are undergoing (Britz et al., 2015; Bikoff et al., 2016). Selectively silencing the entire population of V1 interneurons drastically decreases the speed of locomotor bursts (Gosgnach et al., 2006), although it does not impair ipsilateral coordination.

The V2 population is composed of the V2a and V2b sub-classes. V2a are excitatory interneurons that express the transcription factor Chx10 (Jessell, 2000; Stepien and Arber, 2008). V2a neurons display a wide variety of firing and show diverse projection patterns to V0v, motoneurons and the CPG (Dougherty and Kiehn, 2010; Stepien et al., 2010). They are believed to be involved in right-left alternation (Crone et al., 2008, 2009). In the larval zebrafish, a subpopulation of these cells is involved in rhythm generation (Eklöf-Ljunggren et al., 2012; Ljunggren et al., 2014). In the neonatal mouse spinal cord, a subpopulation of V2a interneurons expresses Shox2. Selectively silencing the entire V2a neurons as well as silencing more specifically Shox2 V2a neurons has no effect on the locomotor rhythm suggesting that a different subpopulation of neurons is involved in rhythm generation. Potential candidates include non-V2a, Shox2+ and Hb9+ interneurons (Dougherty et al., 2013; Caldeira et al., 2017). V2b interneurons are inhibitory and express the transcription factor GATA2/3. Ablating V2b together with V1 interneurons leads to impairment of the ipsilateral coordination between extensor and flexor (Zhang et al., 2014). However, the deficit is not observed when either V1 or V2b is silenced, suggesting some level of functional redundancy.

V3 neurons are defined by the expression of the Sim1 transcription factor and are mainly commissural. This population is highly heterogenous in terms of anatomical distribution and synaptic target. Hence, they are broadly distributed rostro-caudally as well as dorso-ventrally, found in lamina IV-V-VII-VIII and making connections to contralateral motoneurons and interneurons as well as some ipsilateral motoneurons (Zhang et al., 2008). Experiments suppressing V3 activity causes asymmetric locomotor output *in vitro* and uneven gait *in vivo*, suggesting a critical role in interlimb coordination during locomotion (Zhang et al., 2008).

There is no marker to identify the entire population of dI6 interneurons. The population of interneurons originating from this domain is rather subdivided into three subsets expressing either the transcription factor DMRT3, or Wt1, or both. The majority of dI6 interneurons are rhythmically active during locomotion. Dmrt3 mutant display irregular bursting suggesting involvement of Dmrt3+ dI6 interneurons in coordinating locomotor movement (Andersson et al., 2012). The function of the two other subpopulations remains to be determined.

Most of the efforts to genetically identify interneurons in the last two decades have focused on interneurons located in the lumbar spinal cord involved in locomotion with little attention to other types of interneurons extending through multiple spinal segments. More recently, subsets of ispilateral cervical and thoracic long PINs were shown to express Chx10 suggesting a similar developmental origin as that of V2a interneurons (Azim et al., 2014; Ni et al., 2014). A single study investigated the genetic signature of long descending PINs connecting the cervical and lumbar spinal cord and reported virtually no colocalization with Sim1 and En1 but described a discrete subset of long descending PINs that was GATA3 positive, suggesting a common developmental origin to V2b, but not V1 and V3 interneurons (Flynn et al., 2017). Further investigations are warranted to appreciate how genetically identified PINs contribute to the interenlargement propriospinal network.

In summary, there is a great diversity among the genetically identified classes of interneurons. Recent work has focused on their identification based on different transcription factors (Bikoff et al., 2016; Gabitto et al., 2016). This approach will deepen our understanding on the function of spinal interneurons when their physiological signature is related in terms of input – output and function. In the next section, we discuss in parallel the spinal control of locomotion when circuit function is examined with classical electrophysiological methods and pharmacological manipulations, as well as a combination of methods including ablation of spinal neurons in animal and human.

## Spinal Interneuronal Organization of Locomotion

Firing of spinal interneurons is continuously adjusted by peripheral receptors that transmit information from muscle, joint, and skin via the dorsal root ganglia to first order neurons of the dorsal horn. Sensory feedback contributes up to 30% of the neurons net excitatory drive (Macefield et al., 1993), and affects motor output by acting on segmental and supraspinal neural circuits (Mountcastle, 1957; Mackey et al., 2016). Sensory feedback, especially from the hand and foot, project to the spinal cord with a high degree of somatotopical overlap in laminae II–IV (Levinsson et al., 2002), supporting for convergence on common interneurons (Kniffki et al., 1981; Jankowska, 2001). Thus, sensory transformations performed by dorsal horn reflex interneurons are critical in the organization of locomotion, and dorsal horn neurons might be the first key site for interpretation and selection of sensory information.

### Rhythm Generating Interneurons

Spinal interneurons form neural networks that are responsible for the coordinated activity during locomotion. These neural networks, termed as CPGs, can generate rhythmic motor activity in absence of descending and movement-related afferent inputs to the spinal cord (Grillner and Zangger, 1979; Grillner, 1981; Kjaerulff and Kiehn, 1996). In animals, locomotor CPGs have been extensively studied for the last century (reviewed in Rybak et al., 2015; Kiehn, 2016), however, their existence in humans has been debated for many years as it is largely based on similarities between long-latency flexor reflexes to those observed in L-DOPA treated spinalized animals and epidural stimulation induced rhythmic locomotor activity (Bussel et al., 1996; Dimitrijevic et al., 1998). Regardless of our inability to produce rhythmic locomotor activity in absence of descending and afferent inputs in humans, the basic function of spinal interneuronal circuits, that may compose parts of the CPG, are preserved in humans.

### Contribution of Reflex Pathways to Locomotion

#### Group Ia Muscle Afferents Initiated Reflexes

One of the most studied neuronal circuits is the one engaged in the spinal stretch reflex due to its significant contribution to posture and locomotion. The significant contribution of the muscle spindles to stretch-mediated responses was first described in decerebrate cats, in which transection of the dorsal roots completely abolished any muscle response or contraction following knee joint stretching (Liddell and Sherrington, 1924). The reflex origin of the response was recognized in early 19th century but it was not until mid-1950 when its monosynaptic nature and the exclusive contribution of group Ia afferents to the afferent limb of the reflex pathway were demonstrated (Lloyd, 1943a; Eccles and Lundberg, 1958; Lundberg and Winsbury, 1960). These neurophysiological properties were later confirmed in humans. The synaptic Ia afferent transmission at the sacral spinal cord is approximately 0.4 ms in humans (Ertekin et al., 1996). This synaptic delay was evaluated based on (1) surface EMG recordings of the soleus H-reflex following tibial nerve stimulation, (2) surface EMG recordings of motor responses following epidural stimulation at L4-5 or L5-S1, and (3) soleus EMG response recordings following bipolar epidural electrical stimulation (Ertekin et al., 1996). The delay is consistent with the monosynaptic reflex delay reported in humans by Magladery et al. (1951a,b) during which anterior root action potentials were recorded upon tibial nerve stimulation at low intensities, and in cats following electrical stimulation of the group Ia afferents from the dorsal roots (Lloyd, 1943a,b). We should note that the stretch and/or tendon tap reflexes are oligosynaptic and are not purely Ia-mediated in humans. This is based on the delayed onset of excitation in single motor units and long-lasting multiple discharges (25 ms) with tendon percussion, delayed ventral root responses elicited by Achilles tendon tap, and a sacral cord conduction delay of 4.1 ms for the Achilles tendon reflex (Burke et al., 1983, 1984; Ertekin et al., 1995).

The stretch reflex is deeply modulated during locomotion, a phenomenon that has been attributed to central spinal mechanisms. Specifically, it reaches its maximal amplitude before the peak soleus locomotor EMG activity resulting in an increased reflex during the extension phase and decreased reflex during the flexion phase of locomotion in mesencephalic cats (Akazawa et al., 1982). The phase-dependent stretch reflex amplitude modulation is attributed to modulation in the excitability of alpha motoneurons, and modulation produced by the CPG affecting the excitability of motoneurons via premotoneuronal networks. The rhythmic modulation of Ia EPSPs lends support to the involvement of a CPG driving afferent volleys (Schomburg and Behrends, 1978). Similarly to the observations in decerebrate cats, the short-latency soleus stretch and H-reflexes in humans are deeply modulated during walking, with both reflexes increased at heel contact (relative to swing phase), progressively increased in amplitude from mid to late stance phase, decreasing to zero just before or during the stance-to-swing transition phase followed by significant depression during the swing phase and possible increase in late-swing phase (Capaday and Stein, 1986; Sinkjær et al., 1996; Knikou et al., 2009, 2011). At heel contact, proper force absorbtion from the impact is ensured by concomitant eccentric contraction of the tibialis anterior and concentric contraction of the triceps surae, during which the spinal soleus stretch or H-reflex are mostly downregulated. At this phase, it is the quadriceps H-reflex in humans that increases while it remains deeply depressed throughout the remaining step cycle (Dietz et al., 1990; Larsen et al., 2006). The increased quadriceps H-reflex ensures knee joint shock absorption and stretch-mediated shortening of the knee extensors at heel contact and early stance phase. The soleus H-reflex is progressively increased from mid to late stance contributing to the necessary ankle stiffness needed during push off.

The similar modulation pattern of the ankle stretch and H-reflex in humans and mesencephalic cats and humans, attributed to phase-dependent modulation of presynaptic and postsynaptic spinal inhibitory mechanisms and the CPG regulating these spinal neural circuits in consolidation or in absence of supraspinal control (Knikou, 2010), suggests that quadrupedal and bipedal locomotion may have common neural ensembles regardless of the evolution of gait.

#### Contribution of Ia Interneurons and Renshaw Cells

Reciprocal inhibition is the neural basis for relaxation of antagonist muscles during flexion-extension movements. Reciprocal inhibition is centrally controlled in parallel with the corresponding motoneurons to produce a coordinated contraction of flexors and relaxation of extensors and vice versa. During locomotion, the neural control of reciprocal inhibition is not a simple reflexive action but rather the result of complex neural mechanisms at multiple segments on both sides of the spinal cord. Although IaINs and RCs mediating reciprocal and recurrent inhibition do not generate the locomotor rhythm (Pratt and Jordan, 1987), as rhythm generation is generally accepted to depend on glutamatergic excitatory interneurons (see Evidence From Animal Studies Using Molecular Genetic Approaches and Classical Electrophysiological Methods), IaINs and RCs shape and modulate the locomotor pattern. For example, quadriceps IaINs exhibit a decrease in discharge frequency at the end of the extension phase of the step cycle, that coincides with increased rates of firing in extensor RCs (Pratt and Jordan, 1987), while tibialis anterior IaINs are mostly active during the hyperpolarization of extensor motoneurons (Geertsen et al., 2011) during fictive locomotion. RCs are rhythmically active and discharge in phase with the motoneurons from which they are excited (Pratt and Jordan, 1987), consistent with the reported recurrent IPSPs on motoneurons produced by ventral root stimulation during all phases of the step cycle (McCrea et al., 1980). Simultaneous extracellular recordings from IaINs and intracellular recordings from motoneurons during fictive locomotion in spinal cats confirmed that IaINs are active mostly in the inactive (flexion) phase when their target motoneurons are hyperpolarized (Geertsen et al., 2011).

In mice with selective loss of V1 interneurons, some of which differentiate into RCs and IaINs, flexor-extensor alternation during locomotor-like oscillations remains unaffected, suggesting that IaINs and RCs are not responsible for flexor-extensor coordination (Gosgnach et al., 2006). Based on these findings, the authors suggested that IaINs and RCs play a critical role in determining the frequency of the CPG rhythm and in controlling the speed of locomotor movements (Gosgnach et al., 2006). However, current-clamp recordings revealed pathological prolonged periods of motoneurons membrane depolarization, and maintained firing during the depolarized phase resulting in increased step cycle durations and EMG bursts (Gosgnach et al., 2006). Thus, elimination of V1 neurons activity may not be sufficient to prevent the expression of the normal pattern of alternating flexor and extensor activity and right-left alternation. Whether this is due to lack of V1 contributing to coordination or because different classes of neurons are engaged in these mice models remains to be determined. One might assume that this is because reciprocal IaINs are not involved in the flexor-extensor coordination. However, the latter preposition is against the physiological and anatomical connectivity of IaINs in the cat spinal cord (Hultborn et al., 1971a,b, 1976a; Jankowska et al., 2005), and that nearly all RCs receive excitatory or inhibitory inputs during locomotor-like rhythmic activity in isolated mice spinal cords pretreated with NMDA and serotonin (Nishimaru et al., 2006). In an effort to further address the networks involved in flexor-extensor coordination, concomitant blockade of GABA_A_ and glycinergic inhibition from all ventral neurons uncoupled the coordination in vesicular glutamate transporter 2 knockout mice, supporting that IaINs and RCs are sufficient to coordinate the flexor-extensor alternation (Talpalar et al., 2011).

During walking in humans, recurrent inhibition and reciprocal inhibition are modulated in a phase-dependent manner. The recurrent inhibition, exerted from knee extensors on soleus motoneurons, decreases in early stance and increases in late stance phases (Lamy et al., 2008), while the recurrent inhibition from the tibialis anterior onto the biceps femoris during the swing phase is replaced by facilitation in the early stance phase (Marchand-Pauvert and Nielsen, 2002). Further, the amount of reciprocal inhibition exerted from pretibial afferents on soleus motoneurons increases linearly with the contraction level of soleus EMG activity (Capaday et al., 1990), also documented by an increased short-latency soleus H-reflex depression following common peroneal nerve stimulation during the swing phase (Petersen et al., 1999; Mummidisetty et al., 2013). However, the conditioning afferent volley is modulated on top of the test afferent volley modulation during human walking. Thus, the modulation of recurrent inhibition and reciprocal inhibition *per se* during human walking may not exclusively describe the function or contribution of these spinal interneurons to locomotion. In general terms, similarities in function of reciprocal and recurrent inhibitory neural circuits in animals and humans during locomotion do exist.

#### Contribution of Group I Afferents From Extensors to Stance

The duration of the step cycle and EMG locomotor bursts amplitude likely depends on the activity of more than one class of neuron, including those integrating feedback related to loading, stretch, and sensations from the foot. It is also well established that the interneuronally mediated actions of group I extensor afferents on extensor motoneurons reverse from inhibitory to excitatory, not through disinhibition, but an excitatory pathway that becomes active during locomotion. Specifically, stimulation of ankle extensor group I afferents increases the activity of extensor motoneurons during stance or promotes initiation of the extension phase in spinal cats (Conway et al., 1987; Pearson et al., 1992; Gossard et al., 1994). Extracellular recordings suggested that these neurons are located in the intermediate nucleus, while the second-order interneurons are located in lamina VII below the intermediate nucleus (Gossard et al., 1994; Angel et al., 2005).

Further experiments during locomotion in cats, suggested that group I excitatory inputs accounts for 20–50% of the EMG force during the stance phase (Donelan and Pearson, 2004; Donelan et al., 2009), while reflexes evoked by changes in muscle length produce 35% of the force during stance in the cat ankle extensors (Stein et al., 2000). The latter is consistent with the contribution of muscle spindle afferents to locomotion, whereas the activity of the triceps surae in reduced mesencephalic animal preparations is decreased by half during peripheral nerve block of fusimotor axons (Severin, 1970).

In humans, unloading of the ankle extensors in the stance phase reduces the soleus muscle activity by half in early and mid-stance phases (Sinkjær et al., 2000). The reduced soleus EMG activity is attributed to group Ib and/or group II afferents involving IbINs and group II interneurons as the effect is not altered when transmission of Ia afferents is blocked by ischaemia of the leg or when transmission of the antagonistic nerve is blocked by local anesthesia (Sinkjær et al., 2000). The reversal of group I inhibition to group I excitation between ankle synergistic motoneurons reported during fictive locomotion (Conway et al., 1987) was either not evident during walking (Stephens and Yang, 1996) or it was observed in one third of the human subjects being tested (Faist et al., 2006), likely due to the superimposed modulation of the conditioning afferent volley and the non-direct nature of recordings in human locomotor studies.

#### Contribution of Hip Muscle Afferents to Phase Transition

Hip flexor afferents (group Ia and group II from iliopsoas and sartorius muscles) regulate the duration of the stance and/or swing phases and the stance-to-swing and swing-to-stance transition phases in spinal cats (Grillner and Rossignol, 1978; Andersson and Grillner, 1983; Kriellaars et al., 1994; McVea et al., 2005). The swing phase is initiated only when the hip joint passes a threshold extension angle and the contralateral limb is in a position to accept load (Grillner and Rossignol, 1978). Further works provided strong evidence that hip flexor muscle afferents entrain the fictive locomotor pattern in absence of any input from the hip joint or from tendon organ afferents (Pearson and Rossignol, 1991; Kriellaars et al., 1994; Perreault et al., 1995). Entrainment of the locomotor rhythm is also produced from other flexor afferents. For example, tibialis anterior muscle stimulation at high intensities terminates the ongoing flexion and initiates an extension phase (Stecina et al., 2005), while tibialis anterior muscle stretch during the stance phase promotes the onset of flexor burst activity in the ipsilateral leg and of the contralateral extensor activity in spontaneously walking decerebrate cats (Hiebert et al., 1996).

Entrainment of the locomotor rhythm in humans is difficult to demonstrate, but hip afferents adjust the step cycle duration of the contralateral leg (Pang and Yang, 2001), hip extension shortens the stance phase and advances the swing phase, and an inverse relationship between hip position and load at the time of swing phase initiation has also been found in infants during walking (Pang and Yang, 2000). This indicates that these two factors do not act in isolation to regulate the transition, which is similar to that found for reduced animal preparations during fictive locomotion. These neuronal interactions are evident during concomitant imposed hip movements and excitation of Ib afferents that abolishes the soleus H-reflex facilitation commonly observed during hip extension and reverses the soleus H-reflex inhibition during hip flexion to facilitation (Knikou and Rymer, 2002), a pattern largely resembling the soleus H-reflex phase-dependent modulation during waking.

### Cutaneous Afferents Initiated Reflexes and Their Contribution to Locomotion

Cutaneous afferents, especially those arising from the foot region, contribute to the reflex regulation of locomotion likely because of their location as they can sense foot placement, distribution of pressure in metatarsals, and step progression (Rossignol et al., 2006). In freely walking cats, cutaneous afferents discharge mainly around touchdown and toe off, whereas other cutaneous afferents may discharge throughout stance (Loeb et al., 1977). The reflex effects of cutaneous afferents are characterized by a “reversal of actions” that depends on the phase of the step cycle during locomotion. For example, stimulation of the dorsum of the foot during the swing phase enhances flexion, but when the same stimulation is delivered during the stance phase, cutaneous afferents promote extension (Forssberg et al., 1977). Similarly, stimulation of the tibial or sural nerves at low intensities during locomotion in premammillary cats increases or decreases triceps surae EMG burst activity when stimuli are delivered during the extension or flexion phases, respectively (Duysens, 1977). An opposing reflex action is observed when stimulation is delivered at high intensities, supporting the presence of inhibitory and excitatory neural connections from large and small cutaneous afferents (Duysens, 1977).

Cutaneous afferents of the plantar aspect of the foot alter the step cycle duration or even entrain the locomotor rhythm. Stimulation of the plantar aspect of the foot applied during the stance phase increases the amplitude and duration of the ongoing extensor muscle activity, prolongs the stance phase of the ipsilateral limb and/or blocks the contralateral swing initiation, and when stimulation is delivered during the swing phase either prolongs the ongoing flexor activity or shortens the following extensor EMG burst (Duysens and Pearson, 1976; Duysens and Stein, 1978). Evidence for the phasic control of cutaneous reflex pathways during fictive locomotion is available in abundance (Andersson et al., 1978; Quevedo et al., 2005), supporting the existence of a central spinal mechanism regulating circuits integrating cutaneous input during locomotion. The crossed flexor or crossed extensor responses in the intact freely walking and high decerebrate cat (Duysens et al., 1980; Gauthier and Rossignol, 1981), support the notion that interneurons mediating reflex actions of cutaneous afferents may be a part of the CIN system. Further, the ability of cutaneous stimuli to alter the amplitude modulation of PADs and DRPs evoked by muscle group I afferents (Gossard et al., 1989; Ménard et al., 2002, 2003), supports a sophisticated phase-dependent control of cutaneous afferents at presynaptic and premotoneuronal sites during locomotion (Gossard et al., 1989, 1990; Degtyarenko et al., 1996; Burke et al., 2001) that may be controlled by the CPG. The importance of cutaneous input to locomotion is evident in cases in which preservation of minimal cutaneous input is required to promote correct foot placement and weight support in spinalized cats (Bouyer and Rossignol, 2003a,b), by the unstable gait observed in piezo type mechanosensitive ion channel component 2 knockout mice when the mechanotransduction in Merkel cells, muscle spindles, and Golgi tendon organs is prevented (Coste et al., 2010; Ikeda et al., 2014; Woo et al., 2015), and by the improved locomotor recovery when cutaneous feedback is enhanced in spinal animals (Muir and Steeves, 1997; Smith et al., 2006).

In humans at rest, electrical stimulation of low-threshold afferents evokes complex excitatory and inhibitory short- and long-latency reflex responses in multiple leg muscles (Aniss et al., 1992). These complex excitatory and inhibitory reflex responses are not stereotyped but highly modifiable based on the type and phase of the motor task (Burke et al., 1991). During walking, the perceived cutaneous sensation increases at the end of swing phase (Duysens et al., 1995), ensuring appropriate placement of the foot at heel contact and control of the ankle during early stance. Low-intensity stimulation of the sural nerve evokes facilitatory responses in the tibialis anterior muscle at early swing, but tibialis anterior suppression with weak ankle plantar flexion when stimulation is delivered at end-swing (Duysens et al., 1992). The EMG responses of thigh and shank muscles following superficial nerve stimulation are correlated to the kinematics of the knee and ankle joints (Zehr et al., 1997), allowing smooth movement without tripping of the swing phase and correct placement of the foot in order to accept the weight at the beginning of the stance. The reversal of reflex actions is also evident in antagonistic muscles, during which a dominant tibialis anterior response during swing becomes a facilitatory response in triceps surae at equivalent latencies during stance (Duysens et al., 1990). These findings clearly support for complete reflex reversals in the sign of cutaneous reflexes in human, as originally documented in the cat (Forssberg et al., 1975), lending further support for the existence of CPGs engaged in a similar manner in both animals and humans.

### Contribution of Commissural and Propriospinal Interneurons to Limb Coordination

#### Evidence From Animal Studies Using Molecular Genetic Approaches and Classical Electrophysiological Methods

Right-left limb coordination may be controlled by neural networks integrating inputs related to bilateral hip extension and limb loading. This can be accomplished via continuous presynaptic regulation of synaptic transmission from group I, II afferents as well as cutaneous afferents (Gosgnach et al., 2000; Ménard et al., 2003), and actions between neurons integrating synaptic events of flexor reflex afferents to motoneurons bilaterally (Hultborn et al., 1998; Schomburg et al., 1998). These pathways likely involve neurons that ensure appropriate control of bilateral motoneurons of multiple spinal segments in a timely manner. Thus, CINs, which have axons that cross the midline and provide communication between the two halves of the spinal cord regarding motoneuron output, are crucial for the right-left limb coordination (Buchanan and Grillner, 1987; Buchanan and McPherson, 1995; Chédotal, 2014).

The activity of spinal CINs and their importance during fictive locomotion has been described in the isolated spinal cord of the lamprey (Biróet et al., 2008), neonatal mouse (Zhong et al., 2006), and spinal cat (Matsuyama et al., 2004). Most of the V0 and V3 interneurons are commissural. Recent studies utilizing Dbx1 mutant mice lacking V0 interneurons provide clear evidence for the important role of CINs in locomotion. Mice with selective loss of V0_D_ and V0_V_ CINs, which respectively form inhibitory connections with contralateral motoneurons and excitatory connections with V1 interneurons (RC, IaIN and others), exhibit episodes of abnormal locomotion with increased incidence of cocontraction between right and left locomotor activity, while unilateral phasic flexor-extensor activity largely remains normal (Lanuza et al., 2004). In addition, ablation of V0 inhibitory neurons in transgenic mice leads to an activity pattern with no clear right-left alternation at low locomotor speeds, mixed coordination at medium speeds, and alternation at high locomotor speeds (Talpalar et al., 2013). Although not commissural in nature, V2a neurons project to contralateral motoneurons through synaptic connections with ipsilateral CINs V0_V_. Increasing evidence suggests that they directly control the right-left CPG networks in rodents (Crone et al., 2008, 2009; Dougherty and Kiehn, 2010; Zhong et al., 2010). Ablation of V2a neurons, increases the variability of the step cycle (frequency and amplitude), and severely disturbs the right-left leg coordination (Crone et al., 2008).

Silencing V3 CINs in the awake behaving adult mice markedly increases the variability of both stance and swing phases of the step cycle as well as the amplitude of locomotor bursts (Zhang et al., 2008). Modeling studies suggest that V3 CINs produce a progressive increase in the locomotor speed accompanied by sequential changes of gaits (Danner et al., 2016). In mice that have received training with different locomotor tasks, V3 neurons were preferentially activated in swimming, while both dorsal and ventral V3 neurons were actively recruited during running (Borowska et al., 2013), suggesting a task-dependent recruitment of these neurons.

Ablation of V2a or V3 interneurons does not abolish rhythmic activity, although may disrupt right-left alternation (Crone et al., 2008, 2009; Zhang et al., 2008). This is because the major source of rhythm generation is believed to rely on other populations including the Shox2+ non-V2a interneurons (Dougherty et al., 2013) and a small group of interneurons that are Hb9^+^ and display rhythmogenic cellular properties and synchronized firing (Brownstone and Wilson, 2008; Ziskind-Conhaim et al., 2010; Caldeira et al., 2017). Based on the above evidence, CINs are likely responsible for driving right-left coordination but are not critical to rhythm generation (Lanuza et al., 2004; Crone et al., 2008).

In addition to neurons connecting the two halves of the spinal cord, neurons coupling cervical and lumbar spinal segments ensure postural stability during locomotion and forelimb-hindlimb coordination. Classical neurophysiological studies have shown that the EPSPs of hindlimb motoneurons in response to forelimb nerve stimulation are modulated in a phase-dependent manner during fictive locomotion in high spinal cats (Schomburg et al., 1977). Similarly, stimulation over the skin of the metacarpals and metatarsals increases activity of flexors or extensor muscles of the corresponding hindlimb or forelimb in a phase-dependent manner (Miller et al., 1977). Injection of intraspinal rabies virus in cervical and lumbar spinal cords of mice during locomotion produced postural instability and impaired forelimb and hindlimb coordination (Ruder et al., 2016), lending support for a significant contribution of long PINs to postural stability during locomotion.

Genetic identification of neurons connecting the two halves of the spinal cord and caudal with rostral spinal segments, in addition to studies postulating synaptic linkages between these neurons via classical electrophysiological studies, cannot readily be applicable to humans but they do provide an insight into the molecular identity of neurons involved in mammalian locomotor control, and may contribute in the near future to targeted therapies of impaired gait when the spinal neurons that generate locomotion are delineated.

#### Evidence From Human Studies Using Electrophysiological Methods

Human walking requires a fine coordination between the two legs during which flexors or extensors on one side of the body are silent whilst those on the other side are active. This pattern is enabled by CINs being reinforced by mutual inhibition between flexors and extensors on the same side. Contribution of CINs to human walking is clearly evident from the behavior of interlimb spinal reflexes. Stimulation of the tibial nerve at the end of ipsilateral swing phase decreases the contralateral soleus EMG and increases the contralateral gastrocnemius EMG at a longer latency (Gervasio et al., 2013). This effect is consistent with observations in intact cats (Duysens and Loeb, 1980). The inhibition of soleus and subsequent facilitation of gastrocnemius medialis at the end of ipsilateral swing phase may act as a neural coupling mechanism of knee and ankle joints preparing the ankle for heel contact. However, this is not the case for cats since responses in the contralateral soleus muscle are absent upon manifestation of excitatory responses in the contralateral gastrocnemius medialis (Duysens and Loeb, 1980). Mathematical modeling predicted that the presence of short-latency crossed responses in humans is correlated to the activity of muscle spindle secondary afferents (Gervasio et al., 2017), and thus to group II interneurons. The central latency (∼3 ms) of the contralateral soleus motoneurons reflex response suggests for transmission through an oligosynaptic pathway (Hanna-Boutros et al., 2014), while the different intensities required for early and late phases of inhibition are suggestive for involvement of both group I and II afferents (Hanna-Boutros et al., 2014). However, cutaneous afferents are also involved in crossed reflex actions on the basis that stimulation of the superficial peroneal nerve of the left leg at 100 ms before tibial nerve of the right leg modulates the soleus H-reflex excitability in a phase-dependent manner (Suzuki et al., 2016).

When we walk, arms, and legs may move in synchrony or in opposition. Although, interlimb coordination is considered as a residual function of quadrupedal locomotion (Dietz, 2002), evidence suggests that human bipedal locomotion also relies on neural interactions between cervical and lumbar segments of the spinal cord. For example, propriospinally mediated group I inhibition from plantar muscles can potentially decrease excessive extensor reflex activity during the stance phase (Abbruzzese et al., 1996), allowing for a smooth stance-to-swing transition. During treadmill walking, stimulation of the hand and foot evokes interlimb cutaneous reflexes in both the arms and legs that are modulated in a phase-dependent pattern during walking (Haridas and Zehr, 2003). Stimulation of the foot during walking evokes large cutaneous reflexes in shoulder muscles. The reflex responses from hand to foot and from foot to hand are organized in a reciprocal pattern. Foot stimulation evokes inhibition in the ipsilateral posterior deltoid muscle during stance, while facilitation in the contralateral posterior deltoid muscle is present during contralateral stance. Further, the crossed reflex effects for each arm are present at the same step cycle phase as are evident for the leg. For example, inhibition in the ipsilateral and contralateral tibialis anterior muscle is present during late-swing following hand stimulation, consistent with the effects of foot stimulation. The responses to foot stimulation during early swing were proposed to represent the stumbling corrective response, while the responses following hand stimulation were regarded as a protective mechanism in case the hand contacts an obstacle (Haridas and Zehr, 2003). Further, stimulation of the superficial radial nerve of both arms produced a significant soleus H-reflex in the early stance phase that is replaced by facilitation during standing (Suzuki et al., 2016). We may consider that these stimulation-induced responses may not function in the same way during free walking, but the similar muscle activity pattern of arms and legs during walking, cycling, and stepping (Zehr et al., 2007) suggests for strong neural links of arms and legs during locomotor tasks in humans.

### Locomotor Electromyographic Activity

**FIGURE 5:**
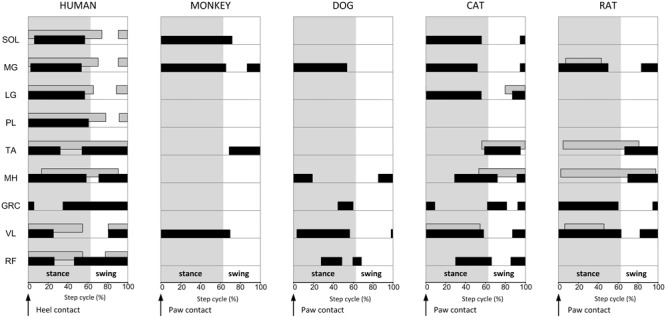
Locomotor electromyographic activity in the intact human, monkey, dog, cat, and rat. Duration of leg/hindlimb muscle activity is shown against normalized step cycle that starts at heel or paw contact; shaded areas mark the stance phase duration. Forward walking is shown as a solid black box whilst backward walking is a patterned box. Absent boxes among muscles is due to the lack of available data. Walking muscle activation patterns adopted and modified from Knikou et al. (2009) and La Scaleia et al. (2014) (human EMG); Courtine et al. (2005) (monkey EMG); Deban et al. (2012) and Goslow et al. (1981) (dog EMG); Buford and Smith (1990) and Yakovenko et al. (2002) (cat EMG); Thota et al. (2005) and Courtine et al. (2009) (rat EMG). SOL, soleus; MG, medialis gastrocnemius; LG, lateral gastrocnemius; PL, peroneus longus; TA, tibialis anterior; MH, medial hamstrings; LH, lateral hamstrings; GRC, gracilis; VL, vastus lateral; RF, rectus femoris.

The phase-dependent modulation of monosynaptic and polysynaptic reflex responses within a limb and between limbs reflects the function of multiple spinal cord interneurons acting as an integrated whole. Neurophysiological evidence suggests that common neural ensembles are preserved in both animal and human. IaINs, RCs, group II interneurons, neurons mediating sensation from the periphery, and neurons crossing the midline of the spinal cord or connecting cervicothoracic and lumbosacral segments are significant contributors to locomotion. Locomotor EMG activity in animals, non-human primates and humans during forward and backward walking (**Figure 5**), undoubtedly demonstrates that motoneuron pools are active at different times of the step cycle across species. For example, the triceps surae/tibialis anterior cocontraction at heel contact in humans is absent in the cat and rat, the hip adductor gracilis muscle activity at early- and late-stance and thought out the swing phase in human is active mostly during the stance phase in the rat and under a multi-phase modulation pattern in the cat (**Figure 5**). Similarly, the knee extensor muscles are active during the early stance phase in human but throughout the stance phase in the dog, cat and rat. These different muscle activation patterns point for participation of additional or different neuronal ensembles in humans compared to animals, as a result of bipedal locomotion. When humans walk on four limbs, the hip flexor and knee extensor muscles are characterized by prolonged bursts (MacLellan et al., 2012), similar to the prolonged pattern of proximal limb muscles in cats (Yakovenko et al., 2002) and monkeys (Courtine et al., 2005). The proximal limb muscle activity coincides with absent activity of plantar flexor muscles during stance (MacLellan et al., 2012), a typical EMG activity for bipedal walking. Interlimb coordination during four-point crawling is manifested as trot-like, pace-like, and mix-limb pairing (Patrick et al., 2009; Gallaher et al., 2011). Similarly, when non-human primates walk bipedally, their gait differs from that of humans, with absent heel contact, absent ankle dorsiflexion after foot contact, heel contact during mid- to late-stance phases, weak push off, more hip flexion, and poorly developed mechanisms of hip abduction, a critical contributor to pelvis and trunk control (Elftman and Manter, 1935; Manter, 1938; Prost, 1967; Grillner, 1981).

The altered EMG activity during bipedal and quadrupedal locomotion suggests a different function of common spinal neural circuitries across species that can potentially be attributed to physiological and anatomical adaptations subserving bipedal gait. For example, heteronymous Ia connections from medialis gastrocnemius to soleus in humans are absent, very small in the baboon, and large in the cat (Eccles et al., 1957b; Pierrot-Deseilligny et al., 1983; Hongo et al., 1984). Further, there are strong heteronymous connections in humans linking muscles crossing multiple joints as evidenced by the effects exerted from gastrocnemius to quadriceps motoneurons via Ia afferents. These neural interactions ensure weight bearing in the stance phase of walking by slowing down the passive ankle dorsiflexion, and thus have been adapted in humans to subserve bipedal gait or unilateral standing. Further, the strong intersegmental coupling stabilizing the vertical trunk and optimizing energy-saving pendulum movements (Ivanenko et al., 2004; Hirasaki et al., 2004) confirms the presence of significant neuronal differences between bipedal and quadrupedal gaits.

## Concluding Remarks

The function of neurons and intraspinal neural circuits testify toward the significant contribution of the spinal cord to locomotion. The spinal cord neural cells in their body, dendrites, and axons, have encoded information needed for local recognition, regulation of synaptic strength, transformation of sensory afferent feedback, and integration of descending inputs. In order to better understand the functional organization and capabilities of the spinal cord, we need to understand how spinal neurons work at multiple levels.

Evidence from animal and human electrophysiological studies support the notion that the basic neural ensembles in which inhibitory ipsilateral interneurons (IaINs, IbINs, RCs), interneurons connecting the two halves of the spinal cord and spinal segments, and interneurons mediating presynaptic inhibiton are preserved across species. When the function of these interneurons is probed at rest, segmental organization of these interneurons with multidirectional synaptic actions on motoneurons and interneurons is largely preserved. During locomotion, the presynaptic inhibitory network, segmental neural organization of Ia INs, multidirectional synaptic actions on motoneurons by group II interneurons, and targeted recurrent inhibition to specific groups of motoneurons are largely similar in animals and humans, while differences include a weaker locomotor Ib facilitatory circuit, more widespread actions of group II afferents, and phase-dependent locomotor muscle activation patterns. Different locomotor EMG activation patterns are likely driven by variations among the distribution pattern of muscle afferents at multiple segmental levels, resulting in altered convergence and interaction between different classes of spinal internreurons and more complex circuits.

In both animals and humans, findings are derived from experiments recording the responses of motoneurons and interneurons following excitation of muscle, joint, and cutaneous afferents. However, during a rhythmic motor task such as locomotion, multiple spinal interneurons are likely activated or silent to some extent simultaneously. This technical limitation may be resolved with the ongoing development of microelectrode arrays able to deliver targeted stimulation to identified neurons and record separately activity from other neurons, afferents and motor axons simultaneously. Further, electrophysiological methods combined with genetic approaches in the developing rodent spinal cords have provided significant advancement in the understanding of the contribution of spinal interneurons to locomotor function (Kiehn, 2011, 2016). However, whether depletion of a group of cells or altered neuronal function of the remaining intact cells or both relates to the altered motor function and locomotion adaptation requires further quantification. Last, the locomotor pattern in reduced animal preparations cannot resemble the gait pattern observed in healthy humans or after a CNS injury that is susceptible to control or modifiability by the cerebellum, brainstem and peripheral feedback (Grillner, 1985). Despite an abundance of evidence on gait recovery by locomotor training in people with spinal cord injury (Smith and Knikou, 2016), the neurophysiological changes with respect to function and interaction of PINs, CINs, and IaINs-IbINs remain poorly understood. In addition, repetitive stimulation of the brain and/or spinal cord (Knikou, 2014; Hofstoetter et al., 2017) can be utilized alone or combined with activity-based therapies to further promote locomotor function recovery in people with pathological gait; however, stimulation protocols are still in their infancy and warrant further investigation since they cannot target a specific neuromodulation pathway.

Understanding the function of spinal interneuronal circuits during mammalian locomotion will assist in delineating the neural mechanisms underlying locomotor behavior and control, and contribute to the development of novel targeted locomotor rehabilitation strategies in cases of impaired bipedal gait in humans. Only by combining molecular genetics, histology, and physiological approaches in animals and humans will we be able to map and better understand how spinal cord neural circuits work, and thus optimize neuromodulation protocols and thereby neurorehabilitation.

## Author Contributions

M-PC and MK wrote the manuscript. LM developed all figures and proofread the manuscript. M-PC, MK, and LM approved the final version of the manuscript before submission.

## Conflict of Interest Statement

The authors declare that the research was conducted in the absence of any commercial or financial relationships that could be construed as a potential conflict of interest.

## Abbreviations

CIN: commissural interneuron
CNS: central nervous system
CPG: central pattern generator
DRP: dorsal root potential
EMG: electromyographic
EPSP: excitatory postsynaptic potential
GABA: γ-aminobutyric acid
IaIN: Ia interneuron
IbIN: Ib interneuron
IPSP: inhibitory postsynaptic potential
NMDA: *N*-methyl-D-aspartate
PAD: primary afferent depolarization
PIN: propriospinal interneuron
RC: Renshaw cell

## References

[B1] Abbruzzese M. Rubino V. Schieppati M. (1996). Task-dependent effects evoked by foot muscle afferents on leg muscle activity in humans. *Electroencephalogr. Clin. Neurophysiol.* 101 339–348. 8761044 10.1016/0924-980x(96)95682-9

[B2] Akazawa K. Aldridge J. W. Steeves J. D. Stein R. B. (1982). Modulation of stretch reflexes during locomotion in the mesencephalic cat. *J. Physiol.* 329 553–567. 10.1113/jphysiol.1982.sp014319 7143259 PMC1224796

[B3] Alstermark B. Kümmel H. (1990a). Transneuronal transport of wheat germ agglutinin conjugated horseradish peroxidase into last order spinal interneurones projecting to acromio- and spinodeltoideus motoneurones in the cat. 1. Location of labelled interneurones and influence of synaptic activity on the transneuronal transport. *Exp. Brain Res.* 80 83–95. 10.1007/BF00228850 1694137

[B4] Alstermark B. Kümmel H. (1990b). Transneuronal transport of wheat germ agglutinin conjugated horseradish peroxidase into last order spinal interneurones projecting to acromio- and spinodeltoideus motoneurones in the cat. 2. Differential labelling of interneurones depending on movement type. *Exp. Brain Res.* 80 96–103. 10.1007/BF00228851 1694138

[B5] Alstermark B. Lindström S. Lundberg A. Sybirska E. (1981). Integration in descending motor pathways controlling the forelimb in the cat. 8. Ascending projection to the lateral reticular nucleus from C3-C4 propriospinal also projecting to forelimb motoneurones. *Exp. Brain Res.* 42 282–298. 10.1007/BF00237495 6263663

[B6] Alstermark B. Lundberg A. Pinter M. Sasaki S. (1987). Subpopulations and functions of long C3-C5 propriospinal neurones. *Brain Res.* 404 395–400. 10.1016/0006-8993(87)91402-8 3567583

[B7] Alvarez F. J. Dewey D. E. Harrington D. A. Fyffe R. E. (1997). Cell-type specific organization of glycine receptor clusters in the mammalian spinal cord. *J. Comp. Neurol.* 379 150–170. 10.1002/(SICI)1096-9861(19970303)379:1<150::AID-CNE10>3.0.CO;2-T 9057118

[B8] Alvarez F. J. Fyffe R. E. (2007). The continuing case for the Renshaw cell. *J. Physiol.* 584 31–45. 10.1113/jphysiol.2007.136200 17640932 PMC2277064

[B9] Alvarez F. J. Jonas P. C. Sapir T. Hartley R. Berrocal M. C. Geiman E. J. (2005). Postnatal phenotype and localization of spinal cord V1 derived interneurons. *J. Comp. Neurol.* 493 177–192. 10.1002/cne.20711 16255029 PMC2997483

[B10] Andersson L. S. Larhammar M. Memic F. Wootz H. Schwochow D. Rubin C. J. (2012). Mutations in DMRT3 affect locomotion in horses and spinal circuit function in mice. *Nature* 488 642–646. 10.1038/nature11399 22932389 PMC3523687

[B11] Andersson O. Forssberg H. Grillner S. Lindquist M. (1978). Phasic gain control of the transmission in cutaneous reflex pathways to motoneurones during ‘fictive’ locomotion. *Brain Res.* 149 503–507. 10.1016/0006-8993(78)90493-6 208712

[B12] Andersson O. Grillner S. (1983). Peripheral control of the cat’s step cycle. II. Entrainment of the central pattern generators for locomotion by sinusoidal hip movements during fictive locomotion. *Acta Physiol. Scand.* 118 229–239. 10.1111/j.1748-1716.1983.tb07267.x 6312752

[B13] Angel M. J. Jankowska E. McCrea D. A. (2005). Candidate interneurones mediating group I disynaptic EPSPs in extensor motoneurones during fictive locomotion in the cat. *J. Physiol.* 563 597–610. 10.1113/jphysiol.2004.076034 15618278 PMC1665583

[B14] Aniss A. M. Gandevia S. C. Burke D. (1992). Reflex responses in active muscles elicited by stimulation of low-threshold afferents from the human foot. *J. Neurophysiol.* 67 1375–1384. 10.1152/jn.1992.67.5.1375 1597720

[B15] Araki T. Eccles J. C. Ito M. (1960). Correlation of the inhibitory post-synaptic potential of motoneurones with the latency and time course of inhibition of monosynaptic reflexes. *J. Physiol.* 154 354–377. 10.1113/jphysiol.1960.sp006584 13683960 PMC1359806

[B16] Arber S. (2012). Motor circuits in action: specification, connectivity, and function. *Neuron* 74 975–989. 10.1016/j.neuron.2012.05.011 22726829

[B17] Azim E. Jiang J. Alstermark B. Jessell T. M. (2014). Skilled reaching relies on a V2a propriospinal internal copy circuit. *Nature* 508 357–363. 10.1038/nature13021 24487617 PMC4230338

[B18] Ballion B. Morin D. Viala D. (2001). Forelimb locomotor generators and quadrupedal locomotion in the neonatal rat. *Eur. J. Neurosci.* 14 1727–1738 10.1046/j.0953-816x.2001.01794.x 11860467

[B19] Bannatyne B. A. Edgley S. A. Hammar I. Jankowska E. Maxwell D. J. (2003). Networks of inhibitory and excitatory commissural interneurons mediating crossed reticulospinal actions. *Eur. J. Neurosci.* 18 2273–2284. 10.1046/j.1460-9568.2003.02973.x 14622188 PMC1971243

[B20] Baret M. Katz R. Lamy J. C. Pénicaud A. Wargon I. (2003). Evidence for recurrent inhibition of reciprocal inhibition from soleus to tibialis anterior in man. *Exp. Brain Res.* 152 133–136. 10.1007/s00221-003-1547-9 12898091

[B21] Bareyre F. M. Kerschensteiner M. Raineteau O. Mettenleiter T. C. Weinmann O. Schwab M. E. (2004). The injured spinal cord spontaneously forms a new intraspinal circuit in adult rats. *Nat. Neurosci.* 7 269–277. 10.1038/nn1195 14966523

[B22] Bautista W. Nagy J. I. Dai Y. McCrea D. A. (2012). Requirement of neuronal connexin36 in pathways mediating presynaptic inhibition of primary afferents in functionally mature mouse spinal cord. *J. Physiol.* 590 3821–3839. 10.1113/jphysiol.2011.225987 22615430 PMC3476635

[B23] Behrends T. Schomburg E. D. Steffens H. (1983a). Facilitatory interaction between cutaneous afferents from low threshold mechanoreceptors and nociceptors in segmental reflex pathways to alpha-motoneurons. *Brain Res.* 260 131–134. 6297679 10.1016/0006-8993(83)90773-4

[B24] Behrends T. Schomburg E. D. Steffens H. (1983b). Group II muscle afferents and low threshold mechanoreceptive skin afferents converging onto interneurons in a common reflex pathway to alpha-motoneurons. *Brain Res.* 265 125–128. 10.1016/0006-8993(83)91341-0 6850313

[B25] Benthall K. N. Hough R. A. McClellan A. D. (2017). Descending propriospinal neurons mediate restoration of locomotor function following spinal cord injury. *J. Neurophysiol.* 117 215–229. 10.1152/jn.00544.2016 27760818 PMC5209543

[B26] Berardelli A. Day B. L. Marsden C. D. Rothwell J. C. (1987). Evidence favouring presynaptic inhibition between antagonist muscle afferents in the human forearm. *J. Physiol.* 391 71–83. 10.1113/jphysiol.1987.sp016726 3443961 PMC1192202

[B27] Berkowitz A. (2010). Multifunctional and specialized spinal interneurons for turtle limb movements. *Ann. N. Y. Acad. Sci.* 1198 119–132. 10.1111/j.1749-6632.2009.05428.x 20536926

[B28] Bikoff J. B. Gabitto M. I. Rivard A. F. Drobac E. Machado T. A. Miri A. (2016). Spinal inhibitory interneuron diversity delineates variant motor microcircuits. *Cell* 165 207–219. 10.1016/j.cell.2016.01.027 26949184 PMC4808435

[B29] Birinyi A. Viszokay K. Wéber I. Kiehn O. Antal M. (2003). Synaptic targets of commissural interneurons in the lumbar spinal cord of neonatal rats. *J. Comp. Neurol.* 461 429–440. 10.1002/cne.10696 12746860

[B30] Biróet Z. Hill R. H. Grillner S. (2008). The activity of spinal commissural interneurons during fictive locomotion in the lamprey. *J. Neurophysiol.* 100 716–722. 10.1152/jn.90206.2008 18509075

[B31] Blatow M. Caputi A. Burnashev N. Monyer H. Rozov A. (2003). Ca2+ buffer saturation underlies paired pulse facilitation in calbindin-D28k-containing terminals. *Neuron* 38 79–88. 10.1016/S0896-6273(03)00196-X 12691666

[B32] Borowska J. Jones C. T. Zhang H. Blacklaws J. Goulding M. Zhang Y. (2013). Functional subpopulations of V3 interneurons in the mature mouse spinal cord. *J. Neurosci.* 33 18553–18565. 10.1523/JNEUROSCI.2005-13.2013 24259577 PMC3894417

[B33] Bouyer L. J. Rossignol S. (2003a). Contribution of cutaneous inputs from the hindpaws to the control of locomotion. I. Intact cats. *J. Neurophysiol.* 90 3625–3639. 10.1152/jn.00496.2003 12944536

[B34] Bouyer L. J. Rossignol S. (2003b). Contribution of cutaneous inputs from the hindpaws to the control of locomotion. II. Spinal cats. *J. Neurophysiol.* 90 3640–3653. 10.1152/jn.00497.2003 12944535

[B35] Bras H. Cavallari P. Jankowska E. Kubin L. (1989). Morphology of midlumbar interneurones relaying information from group II muscle afferents in the cat spinal cord. *J. Comp. Neurol.* 290 1–15. 10.1002/cne.902900102 2592606

[B36] Britz O. Zhang J. Grossmann K. S. Dyck J. Kim J. C. Dymecki S. (2015). A genetically defined asymmetry underlies the inhibitory control of flexor-extensor locomotor movements. *eLife* 4:e04718. 10.7554/eLife.04718 26465208 PMC4604447

[B37] Brockett E. G. Seenan P. G. Bannatyne B. A. Maxwell D. J. (2013). Ascending and descending propriospinal pathways between lumbar and cervical segments in the rat: evidence for a substantial ascending excitatory pathway. *Neuroscience* 240 83–97. 10.1016/j.neuroscience.2013.02.039 23454541

[B38] Brooks V. B. Wilson V. J. (1959). Recurrent inhibition in the cat’s spinal cord. *J. Physiol.* 146 380–391. 10.1113/jphysiol.1959.sp006199 13655237 PMC1356879

[B39] Brown A. G. Fyffe R. E. (1981). Direct observations on the contacts made between Ia afferent fibres and alpha-motoneurones in the cat’s lumbosacral spinal cord. *J. Physiol.* 313 121–140. 10.1113/jphysiol.1981.sp013654 7277213 PMC1274440

[B40] Brownstone R. M. Wilson J. M. (2008). Strategies for delineating spinal locomotor rhythm-generating networks and the possible role of Hb9 interneurones in rhythmogenesis. *Brain Res. Rev.* 57 64–76. 10.1016/j.brainresrev.2007.06.025 17905441 PMC5061561

[B41] Buchanan J. T. Grillner S. (1987). Newly identified ‘glutamate interneurons’ and their role in locomotion in the lamprey spinal cord. *Science* 236 312–314. 10.1126/science.3563512 3563512

[B42] Buchanan J. T. McPherson D. R. (1995). The neuronal network for locomotion in the lamprey spinal cord: evidence for the involvement of commissural interneurons. *J. Physiol. Paris* 89 221–233. 10.1016/0928-4257(96)83638-2 8861820

[B43] Buford J. A. Smith J. L. (1990). Adaptive control for backward quadrupedal walking. II. Hindlimb muscle synergies. *J. Neurophysiol.* 64 756–766. 10.1152/jn.1990.64.3.756 2230922

[B44] Burke D. Dickson H. G. Skuse N. F. (1991). Task-dependent changes in the responses to low-threshold cutaneous afferent volleys in the human lower limb. *J. Physiol.* 432 445–458. 10.1113/jphysiol.1991.sp018393 1886063 PMC1181334

[B45] Burke D. Gandevia S. C. McKeon B. (1983). The afferent volleys responsible for spinal proprioceptive reflexes in man. *J. Physiol.* 339 535–552. 10.1113/jphysiol.1983.sp014732 6887033 PMC1199177

[B46] Burke D. Gandevia S. C. McKeon B. (1984). Monosynaptic and oligosynaptic contributions to human ankle jerk and H-reflex. *J. Neurophysiol.* 52 435–448. 10.1152/jn.1984.52.3.435 6090608

[B47] Burke D. Schiller H. H. (1976). Discharge pattern of single motor units in the tonic vibration reflex of human triceps surae. *J. Neurol. Neurosurg. Psychiatry* 39 729–741. 10.1136/jnnp.39.8.729 956859 PMC492438

[B48] Burke R. (1999). The use of state-dependent modulation of spinal reflexes as a tool to investigate the organization of spinal interneurons. *Exp. Brain Res.* 128 263–277. 10.1007/s002210050847 10501799

[B49] Burke R. E. Degtyarenko A. M. Simon E. S. (2001). Patterns of locomotor drive to motoneurons and last-order interneurons: clues to the structure of the CPG. *J. Neurophysiol.* 86 447–462. 10.1152/jn.2001.86.1.447 11431524

[B50] Burke R. E. Fedina L. Lundberg A. (1971). Spatial synaptic distribution of recurrent and group Ia inhibitory systems in cat spinal motoneurones. *J. Physiol.* 214 305–326. 10.1113/jphysiol.1971.sp009434 5579639 PMC1331838

[B51] Bussel B. Pierrot-Deseilligny E. (1977). Inhibition of human motoneurons, probably of Renshaw origin, elicited by an orthodromic motor discharge. *J. Physiol.* 269 319–339. 10.1113/jphysiol.1977.sp011904 894596 PMC1283715

[B52] Bussel B. Roby-Brami A. Néris O. R. Yakovleff A. (1996). Evidence for a spinal stepping generator in man. *Paraplegia* 34 91–92. 10.1038/sc.1996.15 8835032

[B53] Calancie B. Alexeeva N. Broton J. G. Molano M. R. (2005). Interlimb reflex activity after spinal cord injury in man: strengthening response patterns are consistent with ongoing synaptic plasticity. *Clin. Neurophysiol.* 116 75–86. 10.1016/j.clinph.2004.07.018 15589186

[B54] Caldeira V. Dougherty K. J. Borgius L. Kiehn O. (2017). Spinal Hb9: cre-derived excitatory interneurons contribute to rhythm generation in the mouse. *Sci. Rep.* 7:41369. 10.1038/srep41369 28128321 PMC5269678

[B55] Capaday C. Cody F. W. Stein R. B. (1990). Reciprocal inhibition of soleus motor output in humans during walking and voluntary tonic activity. *J. Neurophysiol.* 64 607–616. 10.1152/jn.1990.64.2.607 2213135

[B56] Capaday C. Stein R. B. (1986). Amplitude modulation of the soleus H-reflex in the human during walking and standing. *J. Neurosci.* 6 1308–1313. 10.1523/JNEUROSCI.06-05-01308.1986 3711981 PMC6568550

[B57] Carpenter D. O. Rudomin P. (1973). The organization of primary afferent depolarization in the isolated spinal cord of the frog. *J. Physiol.* 229 471–493. 10.1113/jphysiol.1973.sp010148 4541991 PMC1350317

[B58] Cavallari P. Edgley S. A. Jankowska E. (1987). Post-synaptic actions of midlumbar interneurones on motoneurones of hindlimb muscles in the cat. *J. Physiol.* 389 675–689. 10.1113/jphysiol.1987.sp016677 3681740 PMC1192101

[B59] Cavallari P. Katz R. Penicaud A. (1992). Pattern of projections of group I afferents from elbow muscles to motoneurones supplying wrist muscles in man. *Exp. Brain Res.* 91 311–319. 10.1007/BF00231664 1459232

[B60] Cazalets J. R. Borde M. Clarac F. (1995). Localization and organization of the central pattern generator for hindlimb locomotion in newborn rat. *J. Neurosci.* 15 4943–4951. 10.1523/JNEUROSCI.15-07-04943 7623124 PMC6577873

[B61] Chaix Y. Marque P. Meunier S. Pierrot-Deseilligny E. Simonetta-Moreau M. (1997). Further evidence for non-monosynaptic group I excitation of motoneurones in the human lower limb. *Exp. Brain Res.* 115 35–46. 10.1007/PL00005683 9224832

[B62] Chédotal A. (2014). Development and plasticity of commissural circuits: from locomotion to brain repair. *Trends Neurosci.* 37 551–562. 10.1016/j.tins.2014.08.009 25220044

[B63] Conta A. C. Stelzner D. J. (2004). Differential vulnerability of propriospinal tract neurons to spinal cord contusion injury. *J. Comp. Neurol.* 479 347–359. 10.1002/cne.20319 15514981

[B64] Conway B. A. Hultborn H. Kiehn O. (1987). Proprioceptive input resets central locomotor rhythm in the spinal cat. *Exp. Brain Res.* 68 643–656. 10.1007/BF00249807 3691733

[B65] Corna S. Grasso M. Nardone A. Schieppati M. (1995). Selective depression of medium-latency leg and foot muscle responses to stretch by an alpha 2-agonist in humans. *J. Physiol.* 484 803–809. 10.1113/jphysiol.1995.sp020705 7623294 PMC1157962

[B66] Coste B. Mathur J. Schmidt M. Earley T. J. Ranade S. Petrus M. J. (2010). Piezo1 and Piezo2 are essential components of distinct mechanically activated cation channels. *Science* 330 55–60. 10.1126/science.1193270 20813920 PMC3062430

[B67] Courtine G. Gerasimenko Y. van den Brand R. Yew A. Musienko P. Zhong H. (2009). Transformation of nonfunctional spinal circuits into functional states after the loss of brain input. *Nat. Neurosci.* 12 1333–1342. 10.1038/nn.2401 19767747 PMC2828944

[B68] Courtine G. Roy R. R. Hodgson J. McKay H. Raven J. Zhong H. (2005). Kinematic and EMG determinants in quadrupedal locomotion of a non-human primate (Rhesus). *J. Neurophysiol.* 93 3127–3145. 10.1152/jn.01073.2004 15647397

[B69] Courtine G. Song B. Roy R. R. Zhong H. Herrmann J. E. Ao Y. (2008). Recovery of supraspinal control of stepping via indirect propriospinal relay connections after spinal cord injury. *Nat. Med.* 14 69–74. 10.1038/nm1682 18157143 PMC2916740

[B70] Cowley K. C. Schmidt B. J. (1997). Regional distribution of the locomotor pattern-generating network in the neonatal rat spinal cord. *J. Neurophysiol.* 77 247–259. 10.1152/jn.1997.77.1.247 9120567

[B71] Cowley K. C. Zaporozhets E. Schmidt B. J. (2010). Propriospinal transmission of the locomotor command signal in the neonatal rat. *Ann. N. Y. Acad. Sci.* 1198 42–53. 10.1111/j.1749-6632.2009.05421.x 20536919

[B72] Crone C. Hultborn H. Jespersen B. Nielsen J. (1987). Reciprocal Ia inhibition between ankle flexors and extensors in man. *J. Physiol.* 389 163–185. 10.1113/jphysiol.1987.sp016652 3681725 PMC1192076

[B73] Crone S. A. Quinlan K. A. Zagoraiou L. Droho S. Restrepo C. E. Lundfald L. (2008). Genetic ablation of V2a ipsilateral interneurons disrupts left-right locomotor coordination in mammalian spinal cord. *Neuron* 60 70–83. 10.1016/j.neuron.2008.08.009 18940589

[B74] Crone S. A. Zhong G. Harris-Warrick R. Sharma K. (2009). In mice lacking V2a interneurons, gait depends on speed of locomotion. *J. Neurosci.* 29 7098–7109. 10.1523/JNEUROSCI.1206-09.2009 19474336 PMC2731420

[B75] Cullheim S. Kellerth J. O. (1978). A morphological study of the axons and recurrent axon collaterals of cat alpha-motoneurones supplying different hind-limb muscles. *J. Physiol.* 281 285–299. 10.1113/jphysiol.1978.sp012422 702381 PMC1282697

[B76] Cullheim S. Kellerth J. O. (1981). Two kinds of recurrent inhibition of cat spinal alpha-motoneurones as differentiated pharmacologically. *J. Physiol.* 312 209–224. 10.1113/jphysiol.1981.sp013624 7264991 PMC1275549

[B77] Danner S. M. Wilshin S. D. Shevtsova N. A. Rybak I. A. (2016). Central control of interlimb coordination and speed-dependent gait expression in quadrupeds. *J. Physiol.* 594 6947–6967. 10.1113/JP272787 27633893 PMC5134391

[B78] Deban S. M. Schilling N. Carrier D. R. (2012). Activity of extrinsic limb muscles in dogs at walk, trot, gallop. *J. Exp. Biol.* 215 287–300. 10.1242/jeb.063230 22189773

[B79] Degtyarenko A. M. Simon E. S. Burke R. E. (1996). Differential modulation of disynaptic cutaneous inhibition and excitation in ankle flexor motoneurons during fictive locomotion. *J. Neurophysiol.* 76 2972–2985. 10.1152/jn.1996.76.5.2972 8930248

[B80] Delwaide P. J. Figiel C. Richelle C. (1977). Effects of postural changes of the upper limb on reflex transmission in the lower limb. *J. Neurol. Neurosurg. Psychiatry* 40 616–621. 10.1136/jnnp.40.6.616 903777 PMC492771

[B81] Dietz V. (2002). Do human bipeds use quadrupedal coordination? *Trends Neurosci.* 25 462–467. 12183207 10.1016/s0166-2236(02)02229-4

[B82] Dietz V. Faist M. Pierrot-Deseilligny E. (1990). Amplitude modulation of the quadriceps H-reflex in the human during the early stance phase of gait. *Exp. Brain Res.* 79 221–224. 10.1007/BF00228893 2311701

[B83] Dimitrijevic M. R. Gerasimenko Y. Pinter M. M. (1998). Evidence for a spinal central pattern generator in humans. *Ann. N. Y. Acad. Sci.* 860 360–376. 10.1111/j.1749-6632.1998.tb09062.x 9928325

[B84] Donelan J. M. McVea D. A. Pearson K. G. (2009). Force regulation of ankle extensor muscle activity in freely walking cats. *J. Neurophysiol.* 101 360–371. 10.1152/jn.90918.2008 19019974

[B85] Donelan J. M. Pearson K. G. (2004). Contribution of force feedback to ankle extensor activity in decerebrate walking cats. *J. Neurophysiol.* 92 2093–2104. 10.1152/jn.00325.2004 15381742

[B86] Dougherty K. J. Kiehn O. (2010). Functional organization of V2a-related locomotor circuits in the rodent spinal cord. *Ann. N. Y. Acad. Sci.* 1198 85–93. 10.1111/j.1749-6632.2010.05502.x 20536923

[B87] Dougherty K. J. Zagoraiou L. Satoh D. Rozani I. Doobar S. Arber S. (2013). Locomotor rhythm generation linked to the output of spinal shox2 excitatory interneurons. *Neuron* 80 920–933. 10.1016/j.neuron.2013.08.015 24267650

[B88] Drew T. Marigold D. S. (2015). Taking the next step: cortical contributions to the control of locomotion. *Curr. Opin. Neurobiol.* 33 25–33. 10.1016/j.conb.2015.01.011 25643847

[B89] Dubuc R. Brocard F. Antri M. Fénelon K. Gariépy J. F. Smetana R. (2008). Initiation of locomotion in lampreys. *Brain Res. Rev.* 57 172–182. 10.1016/j.brainresrev.2007.07.016 17916380

[B90] Dubuc R. Cabelguen J. M. Rossignol S. (1988). Rhythmic fluctuations of dorsal root potentials and antidromic discharges of primary afferents during fictive locomotion in the cat. *J. Neurophysiol.* 60 2014–2036. 10.1152/jn.1988.60.6.2014 3236059

[B91] Dueñas S. Rudomin P. (1988). Excitability changes of ankle extensor group Ia and Ib fibers during fictive locomotion in the cat. *Exp. Brain Res.* 70 15–25. 3402561 10.1007/BF00271842

[B92] Dutton R. C. Carstens M. I. Antognini J. F. Carstens E. (2006). Long ascending propriospinal projections from lumbosacral to upper cervical spinal cord in the rat. *Brain Res.* 1119 76–85. 10.1016/j.brainres.2006.08.063 16996042

[B93] Duysens J. (1977). Reflex control of locomotion as revealed by stimulation of cutaneous afferents in spontaneously walking premammillary cats. *J. Neurophysiol.* 40 737–751. 10.1152/jn.1977.40.4.737 886369

[B94] Duysens J. Loeb G. E. (1980). Modulation of ipsi- and contralateral reflex responses in unrestrained walking cats. *J. Neurophysiol.* 44 1024–1037. 10.1152/jn.1980.44.5.1024 7441320

[B95] Duysens J. Loeb G. E. Weston B. J. (1980). Crossed flexor reflex responses and their reversal in freely walking cats. *Brain Res.* 197 538–542. 10.1016/0006-8993(80)91143-9 7407573

[B96] Duysens J. Pearson K. G. (1976). The role of cutaneous afferents from the distal hindlimb in the regulation of the step cycle of thalamic cats. *Exp. Brain Res.* 24 245–255. 10.1007/BF00235013 1253857

[B97] Duysens J. Stein R. B. (1978). Reflexes induced by nerve stimulation in walking cats with implanted cuff electrodes. *Exp. Brain Res.* 32 213–224. 10.1007/BF00239728 680040

[B98] Duysens J. Tax A. A. Nawijn S. Berger W. Prokop T. Altenmüller E. (1995). Gating of sensation and evoked potentials following foot stimulation during human gait. *Exp. Brain Res.* 105 423–431. 7498396 10.1007/BF00233042

[B99] Duysens J. Tax A. A. Trippel M. Dietz V. (1992). Phase-dependent reversal of reflexly induced movements during human gait. *Exp. Brain Res.* 90 404–414. 10.1007/BF00227255 1397155

[B100] Duysens J. Trippel M. Horstmann G. A. Dietz V. (1990). Gating and reversal of reflexes in ankle muscles during human walking. *Exp. Brain Res.* 82 351–358. 10.1007/BF00231254 2286237

[B101] Eccles J. C. Eccles R. M. Iggo A. Ito M. (1961a). Distribution of recurrent inhibition among motoneurones. *J. Physiol.* 159 479–499. 10.1113/jphysiol.1961.sp006822 13889048 PMC1359546

[B102] Eccles J. C. Eccles R. M. Iggo A. Lundberg A. (1961b). Electrophysiological investigations on Renshaw cells. *J. Physiol.* 159 461–478. 10.1113/jphysiol.1961.sp006821 13889049 PMC1359545

[B103] Eccles J. C. Eccles R. M. Lundberg A. (1957a). Synaptic actions on motoneurones caused by impulses in Golgi tendon organ afferents. *J. Physiol.* 138 227–252. 10.1113/jphysiol.1957.sp005849 13526123 PMC1363042

[B104] Eccles J. C. Eccles R. M. Lundberg A. (1957b). Synaptic actions on motoneurones in relation to the two components of the group I muscle afferent volley. *J. Physiol.* 136 527–546. 10.1113/jphysiol.1957.sp005778 13429518 PMC1358872

[B105] Eccles J. C. Eccles R. M. Magni F. (1961c). Central inhibitory action attributable to presynaptic depolarization produced by muscle afferent volleys. *J. Physiol.* 159 147–166. 10.1113/jphysiol.1961.sp006798 13889050 PMC1359583

[B106] Eccles J. C. Fatt P. Koketsu K. (1954a). Cholinergic and inhibitory synapses in a pathway from motor-axon collaterals to motoneurones. *J. Physiol.* 126 524–562. 10.1113/jphysiol.1954.sp005226 13222354 PMC1365877

[B107] Eccles J. C. Fatt P. Landgren S. (1956). Central pathway for direct inhibitory action of impulses in largest afferent nerve fibres to muscle. *J. Neurophysiol.* 19 75–98. 10.1152/jn.1956.19.1.75 13286723

[B108] Eccles J. C. Fatt P. Landgren S. Winsbury G. J. (1954b). Spinal cord potentials generated by volleys in the large muscle afferents. *J. Physiol.* 125 590–606. 10.1113/jphysiol.1954.sp005183 13212722 PMC1365631

[B109] Eccles J. C. Krnjevic K. (1959). Potential changes recorded inside primary afferent fibres within the spinal cord. *J. Physiol.* 149 250–273. 10.1113/jphysiol.1959.sp006338 13819182 PMC1363088

[B110] Eccles J. C. Schmidt R. F. Willis W. D. (1962). Presynaptic inhibition of the spinal monosynaptic reflex pathway. *J. Physiol.* 161 282–297. 10.1113/jphysiol.1962.sp006886 13889059 PMC1359623

[B111] Eccles R. M. Lundberg A. (1958). Integrative pattern of Ia synaptic actions on motoneurones of hip and knee muscles. *J. Physiol.* 144 271–298. 10.1113/jphysiol.1958.sp006101 13611693 PMC1356742

[B112] Eccles R. M. Lundberg A. (1959). Supraspinal control of interneurones mediating spinal reflexes. *J. Physiol.* 147 565–584. 10.1113/jphysiol.1959.sp006262 13819185 PMC1357103

[B113] Edgley S. A. Jankowska E. (1987). An interneuronal relay for group I and II muscle afferents in the midlumbar segments of the cat spinal cord. *J. Physiol.* 389 647–674. 10.1113/jphysiol.1987.sp016676 3681739 PMC1192100

[B114] Eguibar J. R. Quevedo J. Jiménez I. Rudomin P. (1994). Selective cortical control of information flow through different intraspinal collaterals of the same muscle afferent fiber. *Brain Res.* 643 328–333. 10.1016/0006-8993(94)90042-6 8032927

[B115] Eide A. L. Glover J. Kjaerulff O. Kiehn O. (1999). Characterization of commissural interneurons in the lumbar region of the neonatal rat spinal cord. *J. Comp. Neurol.* 403 332–345. 10.1002/(SICI)1096-9861(19990118)403:3<332::AID-CNE4>3.0.CO;2-R 9886034

[B116] Eklöf-Ljunggren E. Haupt S. Ausborn J. Dehnisch I. Uhlén P. Higashijima S. (2012). Origin of excitation underlying locomotion in the spinal circuit of zebrafish. *Proc. Natl. Acad. Sci. U.S.A.* 109 5511–5516. 10.1073/pnas.1115377109 22431619 PMC3325722

[B117] Elftman H. Manter J. (1935). The evolution of the human foot, with especial reference to the joints. *J. Anat.* 70 56–67. 17104575 PMC1249279

[B118] English A. W. Tigges J. Lennard P. R. (1985). Anatomical organization of long ascending propriospinal neurons in the cat spinal cord. *J. Comp. Neurol.* 240 349–358. 10.1002/cne.902400403 2468691

[B119] Enríquez-Denton M. Nielsen J. Perreault M. C. Morita H. Petersen N. Hultborn H. (2000). Presynaptic control of transmission along the pathway mediating disynaptic reciprocal inhibition in the cat. *J. Physiol.* 526 623–637. 10.1111/j.1469-7793.2000.t01-1-00623.x 10922013 PMC2270037

[B120] Ertekin C. Mungan B. Ertaş M. (1995). Human root and cord potentials evoked by Achilles tendon tap. *Electromyogr. Clin. Neurophysiol.* 35 259–271. 7498070

[B121] Ertekin C. Mungan B. Uludağ B. (1996). Sacral cord conduction time of the soleus H-reflex. *J. Clin. Neurophysiol.* 13 77–83. 10.1097/00004691-199601000-00008 8988288

[B122] Faist M. Hoefer C. Hodapp M. Dietz V. Berger W. Duysens J. (2006). In humans Ib facilitation depends on locomotion while suppression of Ib inhibition requires loading. *Brain Res.* 1076 87–92. 10.1016/j.brainres.2005.12.069 16472783

[B123] Fedina L. Hultborn H. Illert M. (1975). Facilitation from contralateral primary afferents of interneuronal transmission in the Ia inhibitory pathway to motoneurones. *Acta Physiol. Scand.* 94 198–221. 10.1111/j.1748-1716.1975.tb05880.x 1155177

[B124] Fetcho J. R. Higashijima S. McLean D. L. (2008). Zebrafish and motor control over the last decade. *Brain Res. Rev.* 57 86–93. 10.1016/j.brainresrev.2007.06.018 17825423 PMC2237884

[B125] Flynn J. R. Conn V. L. Boyle K. A. Hughes D. I. Watanabe M. Velasquez T. (2017). Anatomical and molecular properties of long descending propriospinal neurons in mice. *Front. Neuroanat.* 11:5 10.3389/fnana.2017.00005 PMC5292581 28220062

[B126] Flynn J. R. Graham B. A. Galea M. P. Callister R. J. (2011). The role of propriospinal interneurons in recovery from spinal cord injury. *Neuropharmacology* 60 809–822. 10.1016/j.neuropharm.2011.01.016 21251920

[B127] Forget R. Hultborn H. Meunier S. Pantieri R. Pierrot-Deseilligny E. (1989a). Facilitation of quadriceps motoneurones by group I afferents from pretibial flexors in man. 2. Changes occurring during voluntary contraction. *Exp. Brain Res.* 78 21–27. 2591514 10.1007/BF00230682

[B128] Forget R. Pantieri R. Pierrot-Deseilligny E. Shindo M. Tanaka R. (1989b). Facilitation of quadriceps motoneurones by group I afferents from pretibial flexors in man. 1. Possible interneuronal pathway. *Exp. Brain Res.* 78 10–20. 2591506 10.1007/BF00230681

[B129] Forssberg H. Grillner S. Rossignol S. (1975). Phase dependent reflex reversal during walking in chronic spinal cats. *Brain Res.* 85 103–107. 10.1016/0006-8993(75)91013-6 1109686

[B130] Forssberg H. Grillner S. Rossignol S. (1977). Phasic gain control of reflexes from the dorsum of the paw during spinal locomotion. *Brain Res.* 132 121–139. 10.1016/0006-8993(77)90710-7 890471

[B131] Fournier E. Meunier S. Pierrot-Deseilligny E. Shindo M. (1986). Evidence for interneuronally mediated Ia excitatory effects to human quadriceps motoneurones. *J. Physiol.* 377 143–169. 10.1113/jphysiol.1986.sp016179 3795085 PMC1182825

[B132] Frank K. Fuortes M. G. F. (1957). Presynaptic and postsynaptic inhibition of monosynaptic reflexes. *Fed. Proc.* 16 39–40.

[B133] Fu T. C. Hultborn H. Larsson R. Lundberg A. (1978). Reciprocal inhibition during the tonic stretch reflex in the decerebrate cat. *J. Physiol.* 284 345–369. 10.1113/jphysiol.1978.sp012544 731548 PMC1282825

[B134] Fyffe R. E. (1990). Evidence for separate morphological classes of Renshaw cells in the cat’s spinal cord. *Brain Res.* 536 301–304. 10.1016/0006-8993(90)90038-D 2085756

[B135] Gabitto M. I. Pakman A. Bikoff J. B. Abbott L. F. Jessell T. M. Paninski L. (2016). Bayesian sparse regression analysis documents the diversity of spinal inhibitory interneurons. *Cell* 165 220–233. 10.1016/j.cell.2016.01.026 26949187 PMC4831714

[B136] Gallaher S. Pollard J. Porter W. L. (2011). Locomotion in restricted space: kinematic and electromyographic analysis of stoopwalking and crawling. *Gait Posture* 33 71–76. 10.1016/j.gaitpost.2010.09.027 20971644

[B137] Gauthier L. Rossignol S. (1981). Contralateral hindlimb responses to cutaneous stimulation during locomotion in high decerebrate cats. *Brain Res.* 207 303–320. 10.1016/0006-8993(81)90366-8 7470911

[B138] Geertsen S. S. Stecina K. Meehan C. F. Nielsen J. B. Hultborn H. (2011). Reciprocal Ia inhibition contributes to motoneuronal hyperpolarisation during the inactive phase of locomotion and scratching in the cat. *J. Physiol.* 589 119–134. 10.1113/jphysiol.2010.199125 21059756 PMC3039264

[B139] Gervasio S. Farina D. Sinkjær T. Mrachacz-Kersting N. (2013). Crossed reflex reversal during human locomotion. *J. Neurophysiol.* 109 2335–2344. 10.1152/jn.01086.2012 23427302

[B140] Gervasio S. Voigt M. Kersting U. G. Farina D. Sinkjær T. Mrachacz-Kersting N. (2017). Sensory feedback in interlimb coordination: contralateral afferent contribution to the short-latency crossed response during human walking. *PLoS One* 12:e0168557. 10.1371/journal.pone.0168557 28060839 PMC5218569

[B141] Giovanelli Barilari M. Kuypers H. G. (1969). Propriospinal fibers interconnecting the spinal enlargements in the cat. *Brain Res.* 14 321–330. 10.1016/0006-8993(69)90113-9 5794910

[B142] Gosgnach S. Bikoff J. B. Dougherty K. J. El Manira A. Lanuza G. M. Zhang Y. (2017). Delineating the diversity of spinal interneurons in locomotor circuits. *J. Neurosci.* 37 10835–10841. 10.1523/JNEUROSCI.1829-17.2017 29118212 PMC6596484

[B143] Gosgnach S. Lanuza G. M. Butt S. J. Saueressig H. Zhang Y. Velasquez T. (2006). V1 spinal neurons regulate the speed of vertebrate locomotor outputs. *Nature* 440 215–219. 10.1038/nature04545 16525473

[B144] Gosgnach S. Quevedo J. Fedirchuk B. McCrea D. A. (2000). Depression of group Ia monosynaptic EPSPs in cat hindlimb motoneurones during fictive locomotion. *J. Physiol.* 526 639–652. 10.1111/j.1469-7793.2000.00639.x 10922014 PMC2270044

[B145] Goslow G. E. Jr. Seeherman H. J. Taylor C. R. McCutchin M. N. Heglund N. C. (1981). Electrical activity and relative length changes of dog limb muscles as a function of speed and gait. *J. Exp. Biol.* 94 15–42. 7310312 10.1242/jeb.94.1.15

[B146] Gossard J. P. Brownstone R. M. Barajon I. Hultborn H. (1994). Transmission in a locomotor-related group Ib pathway from hindlimb extensor muscles in the cat. *Exp. Brain Res.* 98 213–228. 10.1007/BF00228410 8050508

[B147] Gossard J. P. Cabelguen J. M. Rossignol S. (1989). Intra-axonal recordings of cutaneous primary afferents during fictive locomotion in the cat. *J. Neurophysiol.* 62 1177–1188. 10.1152/jn.1989.62.5.1177 2585048

[B148] Gossard J. P. Cabelguen J. M. Rossignol S. (1990). Phase-dependent modulation of primary afferent depolarization in single cutaneous primary afferents evoked by peripheral stimulation during fictive locomotion in the cat. *Brain Res.* 537 14–23. 10.1016/0006-8993(90)90334-8 2085768

[B149] Goulding M. (2009). Circuits controlling vertebrate locomotion: moving in a new direction. *Nat. Rev. Neurosci.* 10 507–518. 10.1038/nrn2608 19543221 PMC2847453

[B150] Granit R. (1950). Reflex self-regulation of muscle contraction and autogenetic inhibition. *J. Neurophysiol.* 13 351–372. 10.1152/jn.1950.13.5.351 14774750

[B151] Granit R. Haase J. Rutledge L. T. (1960). Recurrent inhibition in relation to frequency of firing and limitation of discharge rate of extensor motoneurones. *J. Physiol.* 154 308–328. 10.1113/jphysiol.1960.sp006581 16992068 PMC1359803

[B152] Grillner S. (1981). *Control of Locomotion in Bipeds, Tetrapods, and Fish.* Washington, DC: American Physiological Society 10.1002/cphy.cp010226

[B153] Grillner S. (1985). Neurobiological bases of rhythmic motor acts in vertebrates. *Science* 22 143–149. 10.1126/science.3975635 3975635

[B154] Grillner S. Parker D. El Manira A. (1998). Vertebrate locomotion–a lamprey perspective. *Ann. N. Y. Acad. Sci.* 860 1–18. 10.1111/j.1749-6632.1998.tb09035.x 9928298

[B155] Grillner S. Robertson B. (2015). The basal ganglia downstream control of brainstem motor centres–an evolutionarily conserved strategy. *Curr. Opin. Neurobiol.* 33 47–52. 10.1016/j.conb.2015.01.019 25682058

[B156] Grillner S. Rossignol S. (1978). On the initiation of the swing phase of locomotion in chronic spinal cats. *Brain Res.* 146 269–277. 10.1016/0006-8993(78)90973-3 274169

[B157] Grillner S. Zangger P. (1979). On the central generation of locomotion in the low spinal cat. *Exp. Brain Res.* 34 241–261. 10.1007/BF00235671 421750

[B158] Hanna-Boutros B. Sangari S. Karasu A. Giboin L. S. Marchand-Pauvert V. (2014). Task-related modulation of crossed spinal inhibition between human lower limbs. *J. Neurophysiol.* 111 1865–1876. 10.1152/jn.00838.2013 24501265

[B159] Haridas C. Zehr E. P. (2003). Coordinated interlimb compensatory responses to electrical stimulation of cutaneous nerves in the hand and foot during walking. *J. Neurophysiol.* 90 2850–2861. 10.1152/jn.00531.2003 12853441

[B160] Harrison P. J. Jankowska E. (1985). Sources of input to interneurones mediating group I non-reciprocal inhibition of motoneurones in the cat. *J. Physiol.* 361 379–401. 10.1113/jphysiol.1985.sp015651 3989732 PMC1192865

[B161] Harrison P. J. Jankowska E. (1989). Primary afferent depolarization of central terminals of group II muscle afferents in the cat spinal cord. *J. Physiol.* 411 71–83. 10.1113/jphysiol.1989.sp017561 2614740 PMC1190512

[B162] Hiebert G. W. Whelan P. J. Prochazka A. Pearson K. G. (1996). Contribution of hind limb flexor muscle afferents to the timing of phase transitions in the cat step cycle. *J. Neurophysiol.* 75 1126–1137. 10.1152/jn.1996.75.3.1126 8867123

[B163] Hirasaki E. Ogihara N. Hamada Y. Kumakura H. Nakatsukasa M. (2004). Do highly trained monkeys walk like humans? A kinematic study of bipedal locomotion in bipedally trained Japanese macaques. *J. Hum. Evol.* 46 739–750. 10.1016/j.jhevol.2004.04.004 15183673

[B164] Hochman S. Shreckengost J. Kimura H. Quevedo J. (2010). Presynaptic inhibition of primary afferents by depolarization: observations supporting nontraditional mechanisms. *Ann. N. Y. Acad. Sci.* 1198 140–152. 10.1111/j.1749-6632.2010.05436.x 20536928

[B165] Hofstoetter U. S. Knikou M. Guertin P. A. Minassian K. (2017). Probing the human spinal locomotor circuits by phasic step-induced feedback and by tonic electrical and pharmacological neuromodulation. *Curr. Pharm. Des.* 23 1805–1820. 10.2174/1381612822666161214144655 27981912

[B166] Hongo T. Jankowska E. Ohno T. Sasaki S. Yamashita M. Yoshida K. (1983). The same interneurones mediate inhibition of dorsal spinocerebellar tract cells and lumbar motoneurones in the cat. *J. Physiol.* 342 161–180. 10.1113/jphysiol.1983.sp014845 6631730 PMC1193953

[B167] Hongo T. Lundberg A. Phillips C. G. Thompson R. F. (1984). The pattern of monosynaptic Ia-connections to hindlimb motor nuclei in the baboon: a comparison with the cat. *Proc. R. Soc. Lond. B Biol. Sci.* 221 261–289. 10.1098/rspb.1984.0034 6146138

[B168] Hultborn H. (2006). Spinal reflexes, mechanisms, and concepts: from Eccles to Lundberg and beyond. *Prog. Neurobiol.* 78 215–232. 10.1016/j.pneurobio.2006.04.001 16716488

[B169] Hultborn H. Brownstone R. B. Toth T. I. Gossard J. P. (2004). Key mechanisms for setting the input-output gain across the motoneuron pool. *Prog. Brain Res.* 143 77–95. 10.1016/S0079-6123(03)43008-2 14653153

[B170] Hultborn H. Conway B. A. Gossard J. P. Brownstone R. Fedirchuk B. Schomburg E. D. (1998). How do we approach the locomotor network in the mammalian spinal cord? *Ann. N. Y. Acad. Sci.* 860 70–82. 10.1111/j.1749-6632.1998.tb09039.x 9928302

[B171] Hultborn H. Denton M. E. Wienecke J. Nielsen J. B. (2003). Variable amplification of synaptic input to cat spinal motoneurones by dendritic persistent inward current. *J. Physiol.* 552 945–952. 10.1113/jphysiol.2003.050971 14500771 PMC2343455

[B172] Hultborn H. Illert M. Santini M. (1976a). Convergence on interneurons mediating the reciprocal Ia inhibition of motoneurones. I. Disynaptic Ia inhibition of Ia inhibitory interneurones. *Acta Physiol. Scand.* 96 193–201. 10.1111/j.1748-1716.1976.tb10188.x 1258669

[B173] Hultborn H. Illert M. Santini M. (1976b). Convergence on interneurons mediating the reciprocal Ia inhibition of motoneurones. II. Effects from segmental flexor reflex pathways. *Acta Physiol. Scand.* 96 351–367. 10.1111/j.1748-1716.1976.tb10205.x 1274617

[B174] Hultborn H. Jankowska E. Lindström S. (1971a). Recurrent inhibition of interneurones monosynaptically activated from group Ia afferents. *J. Physiol.* 215 613–636. 4253675 10.1113/jphysiol.1971.sp009488 PMC1331904

[B175] Hultborn H. Jankowska E. Lindström S. Roberts W. (1971b). Neuronal pathway of the recurrent facilitation of motoneurones. *J. Physiol.* 218 495–514. 5124574 10.1113/jphysiol.1971.sp009630 PMC1331808

[B176] Hultborn H. Meunier S. Morin C. Pierrot-Deseilligny E. (1987). Assessing changes in presynaptic inhibition of Ia fibres: a study in man and the cat. *J. Physiol.* 389 729–756. 10.1113/jphysiol.1987.sp016680 3681741 PMC1192104

[B177] Hunt C. C. (1954). Relation of function to diameter in afferent fibers of muscle nerves. *J. Gen. Physiol.* 38 117–131. 10.1085/jgp.38.1.117 13192320 PMC2147477

[B178] Ikeda R. Cha M. Ling J. Jia Z. Coyle D. Gu J. G. (2014). Merkel cells transduce and encode tactile stimuli to drive Aβ-afferent impulses. *Cell* 157 664–675. 10.1016/j.cell.2014.02.026 24746027 PMC4003503

[B179] Illert M. Lundberg A. Tanaka R. (1976). Integration in descending motor pathways controlling the forelimb in the cat. 1. Pyramidal effects on motoneurones. *Exp. Brain Res.* 26 509–519. 10.1007/BF00238824 1010004

[B180] Ivanenko Y. P. Dominici N. Cappellini G. Dan B. Cheron G. Lacquaniti F. (2004). Development of pendulum mechanism and kinematic coordination from the first unsupported steps in toddlers. *J. Exp. Biol.* 207 3797–3810. 10.1242/jeb.01214 15371487

[B181] Jankowska E. (1992). Interneuronal relay in spinal pathways from proprioceptors. *Prog. Neurobiol.* 38 335–378. 10.1016/0301-0082(92)90024-9 1315446

[B182] Jankowska E. (2001). Spinal interneuronal systems: identification, multifunctional character and reconfigurations in mammals. *J. Physiol.* 533 31–40. 10.1111/j.1469-7793.2001.0031b.x 11351010 PMC2278593

[B183] Jankowska E. (2016a). “Spinal interneurons”, in *Neuroscience in the 21st Century* eds Pfaff D. W. Volkow N. D. (New York, NY: Springer Science+Business Media) 1189–1224. 10.1007/978-1-4939-3474-4_34

[B184] Jankowska E. (2016b). “Spinal reflexes”, in *Neuroscience in the 21st Century* eds Pfaff D. W. Volkow N. D. (New York, NY: Springer Science+Business Media) 1599–1621. 10.1007/978-1-4939-3474-4_50

[B185] Jankowska E. Bannatyne B. A. Stecina K. Hammar I. Cabaj A. Maxwell D. J. (2009). Commissural interneurons with input from group I and II muscle afferents in feline lumbar segments: neurotransmitters, projections and target cells. *J. Physiol.* 587 401–418. 10.1113/jphysiol.2008.159236 19047210 PMC2670052

[B186] Jankowska E. Hammar I. (2002). Spinal interneurones; how can studies in animals contribute to the understanding of spinal interneuronal systems in man? *Brain Res. Brain Res. Rev.* 40 19–28. 10.1016/S0165-0173(02)00185-6 12589903

[B187] Jankowska E. Hammar I. Slawinska U. Maleszak K. Edgley S. A. (2003). Neuronal basis of crossed actions from the reticular formation on feline hindlimb motoneurons. *J. Neurosci.* 23 1867–1878. 10.1523/JNEUROSCI.23-05-01867.2003 12629191 PMC1890022

[B188] Jankowska E. Johannisson T. Lipski J. (1981). Common interneurones in reflex pathways from group 1a and 1b afferents of ankle extensors in the cat. *J. Physiol.* 310 381–402. 10.1113/jphysiol.1981.sp013556 7230041 PMC1274747

[B189] Jankowska E. Jukes M. G. Lund S. Lundberg A. (1967). The effect of DOPA on the spinal cord. 6. Half-centre organization of interneurones transmitting effects from the flexor reflex afferents. *Acta Physiol. Scand.* 70 389–402. 10.1111/j.1748-1716.1967.tb03637.x 4294400

[B190] Jankowska E. Krutki P. Matsuyama K. (2005). Relative contribution of Ia inhibitory interneurones to inhibition of feline contralateral motoneurones evoked via commissural interneurones. *J. Physiol.* 568 617–628. 10.1113/jphysiol.2005.088351 16096343 PMC1474749

[B191] Jankowska E. Lindström S. (1971). Morphological identification of Renshaw cells. *Acta Physiol. Scand.* 81 428–430. 10.1111/j.1748-1716.1971.tb04918.x 4101374

[B192] Jankowska E. Lindström S. (1972). Morphology of interneurones mediating Ia reciprocal inhibition of motoneurones in the spinal cord of the cat. *J. Physiol.* 226 805–823. 10.1113/jphysiol.1972.sp010011 4118049 PMC1331178

[B193] Jankowska E. Lundberg A. Robers W. J. Stuart D. (1974). A long propriospinal system with direct effect on motoneurones and on interneurones in the cat lumbosacral cord. *Exp. Brain Res.* 21 169–194. 10.1007/BF00234388 4373265

[B194] Jankowska E. Lundberg A. Stuart D. (1983). Propriospinal control of interneurons in spinal reflex pathways from tendon organs in the cat. *Brain Res.* 261 317–320. 10.1016/0006-8993(83)90636-4 6831214

[B195] Jankowska E. McCrea D. A. (1983). Shared reflex pathways from Ib tendon organ afferents and Ia muscle spindle afferents in the cat. *J. Physiol.* 338 99–111. 10.1113/jphysiol.1983.sp014663 6224005 PMC1197184

[B196] Jankowska E. Noga B. R. (1990). Contralaterally projecting lamina VIII interneurones in middle lumbar segments in the cat. *Brain Res.* 535 327–330. 10.1016/0006-8993(90)91618-Q 2073610

[B197] Jankowska E. Padel Y. Zarzecki P. (1978). Crossed disynaptic inhibition of sacral motoneurones. *J. Physiol.* 285 425–444. 10.1113/jphysiol.1978.sp012580 745104 PMC1281765

[B198] Jankowska E. Roberts W. J. (1972). Synaptic actions of single interneurones mediating reciprocal Ia inhibition of motoneurones. *J. Physiol.* 222 623–642. 10.1113/jphysiol.1972.sp009818 5033026 PMC1331404

[B199] Jankowska E. Slawinska U. Hammar I. (2002). On organization of a neuronal network in pathways from group II muscle afferents in feline lumbar spinal segments. *J. Physiol.* 542 301–314. 10.1113/jphysiol.2001.014076 12096071 PMC2290388

[B200] Jessell T. M. (2000). Neuronal specification in the spinal cord: inductive signals and transcriptional codes. *Nat. Rev. Genet.* 1 20–29. 10.1038/35049541 11262869

[B201] Jordan L. M. Liu J. Hedlund P. B. Akay T. Pearson K. G. (2008). Descending command systems for the initiation of locomotion in mammals. *Brain Res. Rev.* 57 183–191. 10.1016/j.brainresrev.2007.07.019 17928060

[B202] Juvin L. Simmers J. Morin D. (2005). Propriospinal circuitry underlying interlimb coordination in mammalian quadrupedal locomotion. *J. Neurosci.* 25 6025–6035. 10.1523/JNEUROSCI.0696-05.2005 15976092 PMC6724791

[B203] Kiehn O. (2011). Development and functional organization of spinal locomotor circuits. *Curr. Opin. Neurobiol.* 21 100–109. 10.1016/j.conb.2010.09.004 20889331

[B204] Kiehn O. (2016). Decoding the organization of spinal circuits that control locomotion. *Nat. Rev. Neurosci.* 17 224–238. 10.1038/nrn.2016.9 26935168 PMC4844028

[B205] Kiehn O. Dougherty K. J. (2013). “Locomotion: circuits and physiology,” in *Neuroscience in the 21st Century* ed. Pfaff D. W. (New York, NY: Springer) 1209–1236. 10.1007/978-1-4614-1997-6_42

[B206] Kjaerulff O. Kiehn O. (1996). Distribution of networks generating and coordinating locomotor activity in the neonatal rat spinal cord in vitro: a lesion study. *J. Neurosci.* 16 5777–5794. 10.1523/JNEUROSCI.16-18-05777.1996 8795632 PMC6578971

[B207] Kniffki K. D. Schomburg E. D. Steffens H. (1981). Convergence in segmental reflex pathways from fine muscle afferents and cutaneous or group II muscle afferents to (-motoneurones. *Brain Res.* 218 342–346. 10.1016/0006-8993(81)91312-3 7272740

[B208] Knikou M. (2007). Neural coupling between the upper and lower limbs in humans. *Neurosci. Lett.* 416 138–143. 10.1016/j.neulet.2007.01.072 17331647

[B209] Knikou M. (2010). Neural control of locomotion and training-induced plasticity after spinal and cerebral lesions. *Clin. Neurophysiol.* 121 1655–1668. 10.1016/j.clinph.2010.01.039 20427232

[B210] Knikou M. (2012). Plasticity of corticospinal neural control after locomotor training in human spinal cord injury. *Neural Plast.* 2012:254948. 10.1155/2012/254948 22701805 PMC3373155

[B211] Knikou M. (2014). Transpinal and transcortical stimulation alter corticospinal excitability and increase spinal output. *PLoS One* 9:e102313. 10.1371/journal.pone.0102313 25007330 PMC4090164

[B212] Knikou M. Angeli C. A. Ferreira C. K. Harkema S. J. (2009). Soleus H-reflex modulation during body weight support treadmill walking in spinal cord intact and injured subjects. *Exp. Brain Res.* 193 397–407. 10.1007/s00221-008-1636-x 19011843

[B213] Knikou M. Hajela N. Mummidisetty C. K. Xiao M. Smith A. C. (2011). Soleus H-reflex phase-dependent modulation is preserved during stepping within a robotic exoskeleton. *Clin. Neurophysiol.* 122 1396–1404. 10.1016/j.clinph.2010.12.044 21237704

[B214] Knikou M. Rymer Z. (2002). Effects of changes in hip joint angle on H-reflex excitability in humans. *Exp. Brain Res.* 143 149–159. 10.1007/s00221-001-0978-4 11880891

[B215] Kriellaars D. J. Brownstone R. M. Noga B. R. Jordan L. M. (1994). Mechanical entrainment of fictive locomotion in the decerebrate cat. *J. Neurophysiol.* 71 2074–2086. 10.1152/jn.1994.71.6.2074 7931503

[B216] La Scaleia V. Ivanenko Y. P. Zelik K. E. Lacquaniti F. (2014). Spinal motor outputs during step-to-step transitions of diverse human gaits. *Front. Hum. Neurosci.* 8:305. 10.3389/fnhum.2014.00305 24860484 PMC4030139

[B217] Lafleur J. Zytnicki D. Horcholle-Bossavit G. Jami L. (1992). Depolarization of Ib afferent axons in the cat spinal cord during homonymous muscle contraction. *J. Physiol.* 445 345–354. 10.1113/jphysiol.1992.sp018927 1501138 PMC1179985

[B218] Lamotte d’Incamps B. Ascher P. (2008). Four excitatory postsynaptic ionotropic receptors coactivated at the motoneuron-Renshaw cell synapse. *J. Neurosci.* 28 14121–14131. 10.1523/JNEUROSCI.3311-08.2008 19109494 PMC6671456

[B219] Lamy J. C. Iglesias C. Lackmy A. Nielsen J. B. Katz R. Marchand-Pauvert V. (2008). Modulation of recurrent inhibition from knee extensors to ankle motoneurones during human walking. *J. Physiol.* 586 5931–5946. 10.1113/jphysiol.2008.160630 18936080 PMC2655432

[B220] Lanuza G. M. Gosgnach S. Pierani A. Jessell T. M. Goulding M. (2004). Genetic identification of spinal interneurons that coordinate left–right locomotor activity necessary for walking movements. *Neuron* 42 375–386. 10.1016/S0896-6273(04)00249-1 15134635

[B221] Laporte Y. Lloyd D. P. (1952). Nature and significance of the reflex connections established by large afferent fibers of muscular origin. *Am. J. Physiol.* 169 609–621. 10.1152/ajplegacy.1952.169.3.609 14943853

[B222] Larsen B. Mrachacz-Kersting N. Lavoie B. A. Voigt M. (2006). The amplitude modulation of the quadriceps H-reflex in relation to the knee joint action during walking. *Exp. Brain Res.* 170 555–566. 10.1007/s00221-005-0237-1 16331506

[B223] Levinsson A. Holmberg H. Broman J. Zhang M. Schouenborg J. (2002). Spinal sensorimotor transformation: relation between cutaneous somatotopy and a reflex network. *J. Neurosci.* 22 8170–8182. 10.1523/JNEUROSCI.22-18-08170.2002 12223571 PMC6758104

[B224] Liddell E. G. T. Sherrington C. (1924). Reflexes in response to stretch (myotatic reflexes). *Proc. R. Soc. B* 96 212–242. 10.1098/rspb.1924.0023

[B225] Liddell E. G. T. Sherrington C. S. (1925). Recruitment and some other features of reflex inhibition. *Proc. R. Soc. Lond. Ser. B* 97 488–518. 10.1098/rspb.1925.0016

[B226] Ljunggren E. E. Haupt S. Ausborn J. Ampatzis K. El Manira A. (2014). Optogenetic activation of excitatory premotor interneurons is sufficient to generate coordinated locomotor activity in larval zebrafish. *J. Neurosci.* 34 134–139. 10.1523/JNEUROSCI.4087-13.2014 24381274 PMC6608174

[B227] Lloyd D. P. C. (1943a). Conduction and synaptic transmission of the reflex response to stretch in spinal cats. *J. Neurophysiol.* 6 317–326. 10.1152/jn.1943.6.4.317

[B228] Lloyd D. P. C. (1943b). Neuron patterns controlling transmission of ipsilateral hindlimb reflexes in cat. *J. Neurophysiol.* 6 293–315. 10.1152/jn.1943.6.4.293

[B229] Lloyd D. P. C. (1946). Integrative pattern of excitation and inhibition in two neuron reflex arcs. *J. Neurophysiol.* 9 439–444. 10.1152/jn.1946.9.6.439 20274400

[B230] Loeb G. E. Bak M. J. Duysens J. (1977). Long-term unit recording from somatosensory neurons in the spinal ganglia of the freely walking cat. *Science* 197 1192–1194. 10.1126/science.897663 897663

[B231] Lundberg A. Malmgren K. Schomburg E. D. (1987). Reflex pathways from group II muscle afferents. 1. Distribution and linkage of reflex actions to alpha-motoneurones. *Exp. Brain Res.* 65 271–281. 10.1007/BF00236299 3556457

[B232] Lundberg A. Winsbury G. (1960). Selective adequate activation of large afferents from muscle spindles and Golgi tendon organs. *Acta Physiol. Scand.* 49 155–164. 10.1111/j.1748-1716.1960.tb01939.x 14418911

[B233] Macefield V. G. Gandevia S. C. Bigland-Ritchie B. Gorman R. B. Burke D. (1993). The firing rates of human motoneurones voluntarily activated in the absence of muscle afferent feedback. *J. Physiol.* 471 429–443. 10.1113/jphysiol.1993.sp019908 8120815 PMC1143969

[B234] Mackey A. S. Uttaro D. McDonough M. P. Krivis L. I. Knikou M. (2016). Convergence of flexor reflex and corticospinal inputs on tibialis anterior network in humans. *Clin. Neurophysiol.* 127 706–715. 10.1016/j.clinph.2015.06.011 26122072

[B235] MacLellan M. J. Ivanenko Y. P. Cappellini G. Sylos Labini F. Lacquaniti F. (2012). Features of hand-foot crawling behavior in human adults. *J. Neurophysiol.* 107 114–125. 10.1152/jn.00693.2011 21975454

[B236] Magladery J. W. Porter W. E. Park A. M. Teasdall R. D. (1951a). Electrophysiological studies of nerve and reflex activity in normal man. IV. The two-neurone reflex and identification of certain action potentials from spinal roots and cord. *Bull Johns Hopkins Hosp.* 88 499–519. 14839348

[B237] Magladery J. W. Teasdall R. D. Park A. M. Porter W. E. (1951b). Electrophysiological studies of nerve and reflex activity in normal man. V. Excitation and inhibition of two-neurone reflexes by afferent impulses in the same trunk. *Bull Johns Hopkins Hosp.* 88 520–537. 14839349

[B238] Manter J. T. (1938). The dynamics of quadrupedal walking. *J. Exp. Biol.* 15 522–540. 10.1242/jeb.01916 16272240

[B239] Marchand-Pauvert V. Nicolas G. Marque P. Iglesias C. Pierrot-Deseilligny E. (2005). Increase in group II excitation from ankle muscles to thigh motoneurones during human standing. *J. Physiol.* 566 257–271. 10.1113/jphysiol.2005.087817 15860524 PMC1464738

[B240] Marchand-Pauvert V. Nielsen J. B. (2002). Modulation of heteronymous reflexes from ankle dorsiflexors to hamstring muscles during human walking. *Exp. Brain Res.* 142 402–408. 10.1007/s00221-001-0942-3 11819049

[B241] Marque P. Nicolas G. Simonetta-Moreau M. Pierrot-Deseilligny E. Marchand-Pauvert V. (2005). Group II excitations from plantar foot muscles to human leg and thigh motoneurones. *Exp. Brain Res.* 161 486–501. 10.1007/s00221-004-2096-6 15536552

[B242] Martinez M. Delivet-Mongrain H. Leblond H. Rossignol S. (2012). Effect of locomotor training in completely spinalized cats previously submitted to a spinal hemisection. *J. Neurosci.* 32 10961–10970. 10.1523/JNEUROSCI.1578-12.2012 22875930 PMC6621008

[B243] Matsushita M. Ueyama T. (1973). Ventral motor nucleus of the cervical enlargement in some mammals; its specific afferents from the lower cord levels and cytoarchitecture. *J. Comp. Neurol.* 150 33–52. 10.1002/cne.901500103 4722146

[B244] Matsuyama K. Nakajima K. Mori F. Aoki M. Mori S. (2004). Lumbar commissural interneurons with reticulospinal inputs in the cat: morphology and discharge patterns during fictive locomotion. *J. Comp. Neurol.* 474 546–561. 10.1002/cne.20131 15174072

[B245] Mattei B. Schmied A. Vedel J. P. (2003). Recurrent inhibition of wrist extensor motoneurones: a single unit study on a deafferented patient. *J. Physiol.* 549 975–984. 10.1113/jphysiol.2003.039040 12702741 PMC2342996

[B246] Matthews P. B. C. (1991). The human stretch reflex and the motor cortex. *Trends Neurosci.* 14 87–121. 10.1016/0166-2236(91)90064-2 1709536

[B247] McCrea D. A. (1998). Neuronal basis of afferent-evoked enhancement of locomotor activity. *Ann. N. Y. Acad. Sci.* 860 216–225. 10.1111/j.1749-6632.1998.tb09051.x 9928314

[B248] McCrea D. A. Pratt C. A. Jordan L. M. (1980). Renshaw cell activity and recurrent effects on motoneurons during fictive locomotion. *J. Neurophysiol.* 44 475–488. 10.1152/jn.1980.44.3.475 7441311

[B249] McKenna J. E. Prusky G. T. Whishaw I. Q. (2000). Cervical motoneuron topography reflects the proximodistal organization of muscles and movements of the rat forelimb: a retrograde carbocyanine dye analysis. *J. Comp. Neurol.* 419 286–296. 10.1002/(SICI)1096-9861(20000410)419:3<286::AID-CNE2>3.0.CO;2-3 10723005

[B250] McVea D. A. Donelan J. M. Tachibana A. Pearson K. G. (2005). A role for hip position in initiating the swing-to-stance transition in walking cats. *J. Neurophysiol.* 94 3497–3508. 10.1152/jn.00511.2005 16093331

[B251] Ménard A. Leblond H. Gossard J. P. (2002). Sensory integration in presynaptic inhibitory pathways during fictive locomotion in the cat. *J. Neurophysiol.* 88 163–171. 10.1152/jn.2002.88.1.163 12091542

[B252] Ménard A. Leblond H. Gossard J. P. (2003). Modulation of monosynaptic transmission by presynaptic inhibition during fictive locomotion in the cat. *Brain Res.* 964 67–82. 10.1016/S0006-8993(02)04067-2 12573514

[B253] Meunier S. Penicaud A. Pierrot-Deseilligny E. Rossi A. (1990). Monosynaptic Ia excitation and recurrent inhibition from quadriceps to ankle flexors and extensors in man. *J. Physiol.* 423 661–675. 10.1113/jphysiol.1990.sp018046 2388162 PMC1189781

[B254] Meunier S. Pierrot-Deseilligny E. Simonetta M. (1993). Pattern of monosynaptic heteronymous Ia connections in the human lower limb. *Exp. Brain Res.* 96 534–544. 10.1007/BF00234121 8299754

[B255] Meunier S. Pierrot-Deseilligny E. Simonetta-Moreau M. (1994). Pattern of heteronymous recurrent inhibition in the human lower limb. *Exp. Brain Res.* 102 149–159. 10.1007/BF00232447 7895791

[B256] Miller S. Reitsma D. J. van der Meché F. G. (1973). Functional organization of long ascending propriospinal pathways linking lumbo-sacral and cervical segments in the cat. *Brain Res.* 62 169–188. 10.1016/0006-8993(73)90626-4 4765110

[B257] Miller S. Ruit J. B. van der Meché F. G. (1977). Reversal of sign of long spinal reflexes dependent on the phase of the step cycle in the high decerebrate cat. *Brain Res.* 128 447–459. 10.1016/0006-8993(77)90170-6 884493

[B258] Mizuno Y. Tanaka R. Yanagisawa N. (1971). Reciprocal group I inhibition on triceps surae motoneurons in man. *J. Neurophysiol.* 34 1010–1017. 10.1152/jn.1971.34.6.1010 4329961

[B259] Morin C. Pierrot-Deseilligny E. Hultborn H. (1984). Evidence for presynaptic inhibition of muscle spindle Ia afferents in man. *Neurosci. Lett.* 44 137–142. 10.1016/0304-3940(84)90071-5 6231494

[B260] Mountcastle V. B. (1957). Modality and topographic properties of single neurons of cat’s somatic sensory cortex. *J. Neurophysiol.* 20 408–434. 10.1152/jn.1957.20.4.408 13439410

[B261] Muir G. D. Steeves J. D. (1997). Sensorimotor stimulation to improve locomotor recovery after spinal cord injury. *Trends Neurosci.* 20 72–77. 10.1016/S0166-2236(96)10068-0 9023875

[B262] Mummidisetty C. K. Smith A. C. Knikou M. (2013). Modulation of reciprocal and presynaptic inhibition during robotic-assisted stepping in humans. *Clin. Neurophysiol.* 124 557–564. 10.1016/j.clinph.2012.09.007 23046639

[B263] Ni Y. Nawabi H. Liu X. Yang L. Miyamichi K. Tedeschi A. (2014). Characterization of long descending premotor propriospinal neurons in the spinal cord. *J. Neurosci.* 34 9404–9417. 10.1523/JNEUROSCI.1771-14.2014 25009272 PMC4468139

[B264] Nishimaru H. Restrepo C. E. Kiehn O. (2006). Activity of Renshaw cells during locomotor-like rhythmic activity in the isolated spinal cord of neonatal mice. *J. Neurosci.* 26 5320–5328. 10.1523/JNEUROSCI.5127-05.2006 16707784 PMC6675298

[B265] Nishimaru H. Restrepo C. E. Ryge J. Yanagawa Y. Kiehn O. (2005). Mammalian motor neurons corelease glutamate and acetylcholine at central synapses. *Proc. Natl. Acad. Sci. U.S.A.* 102 5245–5249. 10.1073/pnas.0501331102 15781854 PMC555035

[B266] Pang M. Y. Yang J. F. (2000). The initiation of the swing phase in human infant stepping: importance of hip position and leg loading. *J. Physiol.* 528 389–404. 10.1111/j.1469-7793.2000.00389.x 11034628 PMC2270131

[B267] Pang M. Y. Yang J. F. (2001). Interlimb co-ordination in human infant stepping. *J. Physiol.* 533 617–625. 10.1111/j.1469-7793.2001.0617a.x 11389217 PMC2278628

[B268] Patrick S. K. Noah J. A. Yang J. F. (2009). Interlimb coordination in human crawling reveals similarities in development and neural control with quadrupeds. *J. Neurophysiol.* 101 603–613. 10.1152/jn.91125.2008 19036860 PMC2657078

[B269] Pearson K. G. Ramirez J. M. Jiang W. (1992). Entrainment of the locomotor rhythm by group Ib afferents from ankle extensor muscles in spinal cats. *Exp. Brain Res.* 90 557–566. 10.1007/BF00230939 1426112

[B270] Pearson K. G. Rossignol S. (1991). Fictive motor patterns in chronic spinal cats. *J. Neurophysiol.* 66 1874–1887. 10.1152/jn.1991.66.6.1874 1812222

[B271] Perreault M. C. Angel M. J. Guertin P. McCrea D. A. (1995). Effects of stimulation of hindlimb flexor group II afferents during fictive locomotion in the cat. *J. Physiol.* 487 211–220. 10.1113/jphysiol.1995.sp020872 7473250 PMC1156610

[B272] Petersen N. Morita H. Nielsen J. (1999). Modulation of reciprocal inhibition between ankle extensors and flexors during walking in man. *J. Physiol.* 520 605–619. 10.1111/j.1469-7793.1999.00605.x 10523426 PMC2269590

[B273] Pierrot-Deseilligny E. Bergego C. Mazieres L. (1983). Reflex control of bipedal gait in man. *Adv. Neurol.* 39 699–716. 6660117

[B274] Pierrot-Deseilligny E. Burke D. (2005). *The Circuitry of the Human Spinal Cord: Its Role in Motor Control and Movement Disorders.* Cambridge: Cambridge University Press 10.1017/CBO9780511545047

[B275] Pierrot-Deseilligny E. Katz R. Morin C. (1979). Evidence of Ib inhibition in human subjects. *Brain Res.* 166 176–179. 10.1016/0006-8993(79)90660-7 421149

[B276] Pocratsky A. M. Burke D. A. Morehouse J. R. Beare J. E. Riegler A. S. Tsoulfas P. (2017). Reversible silencing of lumbar spinal interneurons unmasks a task-specific network for securing hindlimb alternation. *Nat. Commun.* 8:1963. 10.1038/s41467-017-02033-x 29213073 PMC5719045

[B277] Powers R. K. Binder M. D. (1985). Determination of afferent fibers mediating oligosynaptic group I input to cat medial gastrocnemius motoneurons. *J. Neurophysiol.* 53 518–529. 10.1152/jn.1985.53.2.518 3156971

[B278] Pratt C. A. Jordan L. M. (1987). Ia inhibitory interneurons and Renshaw cells as contributors to the spinal mechanisms of fictive locomotion. *J. Neurophysiol.* 57 56–71. 10.1152/jn.1987.57.1.56 3559681

[B279] Prost J. H. (1967). Bipedalism of man and gibbon compared using estimates of joint motion. *Am. J. Phys. Anthrop.* 26 135–148. 10.1002/ajpa.1330260205

[B280] Quevedo J. Eguibar J. R. Lomeli J. Rudomin P. (1997). Patterns of connectivity of spinal interneurons with single muscle afferents. *Exp. Brain Res.* 115 387–402. 10.1007/PL00005709 9262194

[B281] Quevedo J. Stecina K. Gosgnach S. McCrea D. A. (2005). Stumbling corrective reaction during fictive locomotion in the cat. *J. Neurophysiol.* 94 2045–2052. 10.1152/jn.00175.2005 15917325

[B282] Quinlan K. A. Kiehn O. (2007). Segmental, synaptic actions of commissural interneurons in the mouse spinal cord. *J. Neurosci.* 27 6521–6530. 10.1523/JNEUROSCI.1618-07.2007 17567813 PMC6672441

[B283] Rastad J. Gad P. Jankowska E. McCrea D. Westman J. (1990). Light microscopical study of dendrites and perikarya of interneurones mediating la reciprocal inhibition of cat lumbar alpha-motoneurones. *Anat. Embryol.* 181 381–388. 10.1007/BF00186910 2346230

[B284] Reed W. R. Shum-Siu A. Onifer S. M. Magnuson D. S. (2006). Inter-enlargement pathways in the ventrolateral funiculus of the adult rat spinal cord. *Neuroscience* 142 1195–1207. 10.1016/j.neuroscience.2006.07.017 16938403 PMC3741649

[B285] Reed W. R. Shum-Siu A. Whelan A. Onifer S. M. Magnuson D. S. (2009). Anterograde labeling of ventrolateral funiculus pathways with spinal enlargement connections in the adult rat spinal cord. *Brain Res.* 1302 76–84. 10.1016/j.brainres.2009.09.049 19766612 PMC2783768

[B286] Renshaw B. (1942). Effects of presynaptic volleys on spread of impulses over the soma of the motoneurones. *J. Neurophysiol.* 5 235–243. 10.1152/jn.1942.5.3.235

[B287] Renshaw B. (1946). Central effects of centripetal impulses in axons of spinal ventral roots. *J. Neurophysiol.* 9 191–204. 10.1152/jn.1946.9.3.191 21028162

[B288] Roberts A. Soffe S. R. Wolf E. S. Yoshida M. Zhao F. Y. (1998). Central circuits controlling locomotion in young frog tadpoles. *Ann. N. Y. Acad. Sci.* 860 19–34. 10.1111/j.1749-6632.1998.tb09036.x 9928299

[B289] Rossignol S. Dubuc R. Gossard J. P. (2006). Dynamic sensorimotor interactions in locomotion. *Physiol. Rev.* 86 89–154. 10.1152/physrev.00028.2005 16371596

[B290] Ruder L. Takeoka A. Arber S. (2016). Long-distance descending spinal neurons ensure quadrupedal locomotor stability. *Neuron* 92 1063–1078. 10.1016/j.neuron.2016.10.032 27866798

[B291] Rudomin P. (1990). Presynaptic inhibition of muscle spindle and tendon organ afferents in the mammalian spinal cord. *Trends Neurosci.* 13 499–505. 10.1016/0166-2236(90)90084-N 1703681

[B292] Rudomin P. (2002). Selectivity of the central control of sensory information in the mammalian spinal cord. *Adv. Exp. Med. Biol.* 508 157–170. 10.1007/978-1-4615-0713-0_19 12171106

[B293] Rudomin P. (2009). In search of lost presynaptic inhibition. *Exp. Brain Res.* 196 139–151. 10.1007/s00221-009-1758-9 19322562

[B294] Rudomin P. Lomelí J. Quevedo J. (2004). Differential modulation of primary afferent depolarization of segmental and ascending intraspinal collaterals of single muscle afferents in the cat spinal cord. *Exp. Brain Res.* 156 377–391. 10.1007/s00221-003-1788-7 14985894

[B295] Rudomin P. Schmidt R.F. (1999). Presynaptic inhibition in the vertebrate spinal cord revisited. *Exp. Brain Res.* 129 1–37. 10.1007/s002210050933 10550500

[B296] Ryall R. W. (1970). Renshaw cell mediated inhibition of Renshaw cells: patterns of excitation and inhibition from impulses in motor axon collaterals. *J. Neurophysiol.* 33 257–270. 10.1152/jn.1970.33.2.257 4313286

[B297] Rybak I. A. Dougherty K. J. Shevtsova N. A. (2015). Organization of the mammalian locomotor CPG: review of computational model and circuit architectures based on genetically identified spinal interneurons. *eNeuro* 2:ENEURO.0069-15.2015. 10.1523/ENEURO.0069-15.2015 26478909 PMC4603253

[B298] Sarica Y. Ertekin C. (1985). Descending lumbosacral cord potentials (DLCP) evoked by stimulation of the median nerve. *Brain Res.* 325 299–301. 10.1016/0006-8993(85)90327-0 3978422

[B299] Schieppati M. Nardone A. (1999). Group II spindle afferent fibers in humans: their possible role in the reflex control of stance. *Prog. Brain Res.* 123 461–472. 10.1016/S0079-6123(08)62882-4 10635742

[B300] Schieppati M. Nardone A. Siliotto R. Grasso M. (1995). Early and late stretch responses of human foot muscles induced by perturbation of stance. *Exp. Brain Res.* 105 411–422. 7498395 10.1007/BF00233041

[B301] Schneider S. P. Fyffe R. E. (1992). Involvement of GABA and glycine in recurrent inhibition of spinal motoneurons. *J. Neurophysiol.* 68 397–406. 10.1152/jn.1992.68.2.397 1326603

[B302] Schomburg E. D. Behrends H. B. (1978). The possibility of phase-dependent monosynaptic and polysynaptic is excitation to homonymous motoneurones during fictive locomotion. *Brain Res.* 143 533–537. 10.1016/0006-8993(78)90363-3 647377

[B303] Schomburg E. D. Petersen N. Barajon I. Hultborn H. (1998). Flexor reflex afferents reset the step cycle during fictive locomotion in the cat. *Exp. Brain Res.* 122 339–350. 10.1007/s002210050522 9808307

[B304] Schomburg E. D. Roesler J. Meinck H. M. (1977). Phase-dependent transmission in the excitatory propriospinal reflex pathway from forelimb afferents to lumbar motoneurones during fictive locomotion. *Neurosci. Lett.* 4 249–252. 10.1016/0304-3940(77)90187-2 19604953

[B305] Severin F. V. (1970). Role of the gamma-motor system in activation of extensor alpha-motor neurons during controlled locomotion. *Biofizika* 15 1096–1102. 5482663

[B306] Sherrington C. S. (1907). On reciprocal innervation of antagonistic muscle – Tenth note. *Proc. R. Soc. Lond. Ser.* 79 337–349. 10.1098/rspb.1907.0026

[B307] Sherrington C. S. (1910). Flexion-reflex of the limb, crossed extension-reflex, and reflex stepping and standing. *J. Physiol.* 40 28–121. 10.1113/jphysiol.1910.sp001362 16993027 PMC1533734

[B308] Sherrington C. S. Laslett E. E. (1903). Observations on some spinal reflexes and the interconnection of spinal segments. *J. Physiol.* 29 58–96. 10.1113/jphysiol.1903.sp000946 16992657 PMC1540608

[B309] Simonetta-Moreau M. Marque P. Marchand-Pauvert V. Pierrot-Deseilligny E. (1999). The pattern of excitation of human lower limb motoneurones by probable group II muscle afferents. *J. Physiol.* 517 287–300. 10.1111/j.1469-7793.1999.0287z.x 10226166 PMC2269311

[B310] Sinkjær T. Andersen J. B. Ladouceur M. Christensen L. O. Nielsen J. B. (2000). Major role for sensory feedback in soleus EMG activity in the stance phase of walking in man. *J. Physiol.* 523 817–827. 10.1111/j.1469-7793.2000.00817.x 10718758 PMC2269822

[B311] Sinkjær T. Andersen J. B. Larsen B. (1996). Soleus stretch reflex modulation during gait in humans. *J. Neurophysiol.* 76 1112–1120. 10.1152/jn.1996.76.2.1112 8871224

[B312] Smith A. C. Knikou M. (2016). A review on locomotor training after spinal cord injury: reorganization of spinal neuronal circuits and recovery of motor function. *Neural Plast.* 2016:1216258. 10.1155/2016/1216258 27293901 PMC4879237

[B313] Smith R. R. Shum-Siu A. Baltzley R. Bunger M. Baldini A. Burke D. A. (2006). Effects of swimming on functional recovery after incomplete spinal cord injury in rats. *J. Neurotrauma* 23 908–919. 10.1089/neu.2006.23.908 16774475 PMC2831776

[B314] Soteropoulos D. S. Edgley S. A. Baker S. N. (2013). Spinal commissural connections to motoneurons controlling the primate hand and wrist. *J. Neurosci.* 33 9614–9625. 10.1523/JNEUROSCI.0269-13.2013 23739958 PMC3951829

[B315] Stecina K. Quevedo J. McCrea D. A. (2005). Parallel reflex pathways from flexor muscle afferents evoking resetting and flexion enhancement during fictive locomotion and scratch in the cat. *J. Physiol.* 569 275–290. 10.1113/jphysiol.2005.095505 16141269 PMC1464219

[B316] Stein R. B. Misiaszek J. E. Pearson K. G. (2000). Functional role of muscle reflexes for force generation in the decerebrate walking cat. *J. Physiol.* 525 781–791. 10.1111/j.1469-7793.2000.00781.x 10856129 PMC2269981

[B317] Stephens M. J. Yang J. F. (1996). Short latency, non-reciprocal group I inhibition is reduced during the stance phase of walking in humans. *Brain Res.* 743 24–31. 10.1016/S0006-8993(96)00977-8 9017226

[B318] Stepien A. E. Arber S. (2008). Probing the locomotor conundrum: descending the ‘V’ interneuron ladder. *Neuron* 60 1–4. 10.1016/j.neuron.2008.09.030 18940581

[B319] Stepien A. E. Tripodi M. Arber S. (2010). Monosynaptic rabies virus reveals premotor network organization and synaptic specificity of cholinergic partition cells. *Neuron* 68 456–472. 10.1016/j.neuron.2010.10.019 21040847

[B320] Sterling P. Kuypers H. G. (1968). Anatomical organization of the brachial spinal cord of the cat. 3. The propriospinal connections. *Brain Res.* 7 419–443. 10.1016/0006-8993(68)90008-5 5639606

[B321] Stokke M. F. Nissen U. V. Glover J. C. Kiehn O. (2002). Projection patterns of commissural interneurons in the lumbar spinal cord of the neonatal rat. *J. Comp. Neurol.* 446 349–359. 10.1002/cne.10211 11954034

[B322] Stuart G. J. Redman S. J. (1990). Voltage dependence of Ia reciprocal inhibitory currents in cat spinal motoneurones. *J. Physiol.* 420 111–125. 10.1113/jphysiol.1990.sp017903 2324981 PMC1190040

[B323] Stubbs P. W. Mrachacz-Kersting N. (2009). Short-latency crossed inhibitory responses in the human soleus muscle. *J. Neurophysiol.* 102 3596–3605. 10.1152/jn.00667.2009 19812287

[B324] Stubbs P. W. Nielsen J. F. Sinkjær T. Mrachacz-Kersting N. (2011a). Crossed spinal soleus muscle communication demonstrated by H-reflex conditioning. *Muscle Nerve* 43 845–850. 10.1002/mus.21964 21607968

[B325] Stubbs P. W. Nielsen J. F. Sinkjær T. Mrachacz-Kersting N. (2011b). Phase modulation of the short-latency crossed spinal response in the human soleus muscle. *J. Neurophysiol.* 105 503–511. 10.1152/jn.00786.2010 21106895

[B326] Suzuki S. Nakajima T. Futatsubashi G. Mezzarane R. A. Ohtsuka H. Ohki Y. (2016). Phase-dependent reversal of the crossed conditioning effect on the soleus Hoffmann reflex from cutaneous afferents during walking in humans. *Exp. Brain Res.* 234 617–626. 10.1007/s00221-015-4463-x 26573576

[B327] Talpalar A. E. Bouvier J. Borgius L. Fortin G. Pierani A. Kiehn O. (2013). Dual-mode operation of neuronal networks involved in left-right alternation. *Nature* 500 85–88. 10.1038/nature12286 23812590

[B328] Talpalar A. E. Endo T. Löw P. Borgius L. Hägglund M. Dougherty K. J. (2011). Identification of minimal neuronal networks involved in flexor-extensor alternation in the mammalian spinal cord. *Neuron* 71 1071–1084. 10.1016/j.neuron.2011.07.011 21943604

[B329] Thota A. K. Watson S. C. Knapp E. Thompson B. Jung R. (2005). Neuromechanical control of locomotion in the rat. *J. Neurotrauma* 22 442–465. 10.1089/neu.2005.22.442 15853462

[B330] Trank T. V. Turkin V. V. Hamm T. M. (1999). Organization of recurrent inhibition and facilitation in motoneuron pools innervating dorsiflexors of the cat hindlimb. *Exp. Brain Res.* 125 344–352. 10.1007/s002210050690 10229025

[B331] Turkin V. V. Monroe K. S. Hamm T. M. (1998). Organization of recurrent inhibition and facilitation in motor nuclei innervating ankle muscles of the cat. *J. Neurophysiol.* 79 778–790. 10.1152/jn.1998.79.2.778 9463441

[B332] Uchiyama T. Windhorst U. (2007). Effects of spinal recurrent inhibition on motoneuron short-term synchronization. *Biol. Cybern.* 96 561–575. 10.1007/s00422-007-0151-7 17431664

[B333] Vavrek R. Girgis J. Tetzlaff W. Hiebert G. W. Fouad K. (2006). BDNF promotes connections of corticospinal neurons onto spared descending interneurons in spinal cord injured rats. *Brain* 129 1534–1545. 10.1093/brain/awl087 16632552

[B334] Wenner P. O’Donovan M. J. Matise M. P. (2000). Topographical and physiological characterization of interneurons that express engrailed-1 in the embryonic chick spinal cord. *J. Neurophysiol.* 84 2651–2657. 10.1152/jn.2000.84.5.2651 11068006

[B335] Willis W. D. (2006). John Eccles’ studies of spinal cord presynaptic inhibition. *Prog. Neurobiol.* 78 189–214. 10.1016/j.pneurobio.2006.02.007 16650518

[B336] Woo S. H. Lukacs V. de Nooij J. C. Zaytseva D. Criddle C. R. Francisco A. (2015). Piezo2 is the principal mechanotransduction channel for proprioception. *Nat. Neurosci.* 18 1756–1762. 10.1038/nn.416210.1038/nn.4162 26551544 PMC4661126

[B337] Yakovenko S. Mushahwar V. VanderHorst V. Holstege G. Prochazka A. (2002). Spatiotemporal activation of lumbosacral motoneurons in the locomotor step cycle. *J. Neurophysiol.* 87 1542–1553. 10.1152/jn.00479.2001 11877525

[B338] Zagoraiou L. Akay T. Martin J. F. Brownstone R. M. Jessell T. M. Miles G. B. (2009). A cluster of cholinergic premotor interneurons modulates mouse locomotor activity. *Neuron* 64 645–662. 10.1016/j.neuron.2009.10.017 20005822 PMC2891428

[B339] Zaporozhets E. Cowley K. C. Schmidt B. J. (2006). Propriospinal neurons contribute to bulbospinal transmission of the locomotor command signal in the neonatal rat spinal cord. *J. Physiol.* 572 443–458. 10.1113/jphysiol.2005.102376 16469789 PMC1779678

[B340] Zehr E. P. Balter J. E. Ferris D. P. Hundza S. R. Loadman P. M. Stoloff R. H. (2007). Neural regulation of rhythmic arm and leg movement is conserved across human locomotor tasks. *J. Physiol.* 582 209–227. 10.1113/jphysiol.2007.133843 17463036 PMC2075277

[B341] Zehr E. P. Collins D. F. Chua R. (2001). Human interlimb reflexes evoked by electrical stimulation of cutaneous nerves innervating the hand and foot. *Exp. Brain Res.* 140 495–504. 10.1007/s002210100857 11685403

[B342] Zehr E. P. Komiyama T. Stein R. B. (1997). Cutaneous reflexes during human gait: electromyographic and kinematic responses to electrical stimulation. *J. Neurophysiol.* 77 3311–3325. 10.1152/jn.1997.77.6.3311 9212277

[B343] Zhang J. Lanuza G. M. Britz O. Wang Z. Siembab V. C. Zhang Y. (2014). V1 and v2b interneurons secure the alternating flexor-extensor motor activity mice require for limbed locomotion. *Neuron* 82 138–150. 10.1016/j.neuron.2014.02.013 24698273 PMC4096991

[B344] Zhang Y. Narayan S. Geiman E. Lanuza G. M. Velasquez T. Shanks B. (2008). V3 spinal neurons establish a robust and balanced locomotor rhythm during walking. *Neuron* 60 84–96. 10.1016/j.neuron.2008.09.027 18940590 PMC2753604

[B345] Zhong G. Díaz-Ríos M. Harris-Warrick R. M. (2006). Intrinsic and functional differences among commissural interneurons during fictive locomotion and serotonergic modulation in the neonatal mouse. *J. Neurosci.* 26 6509–6517. 10.1523/JNEUROSCI.1410-06.2006 16775138 PMC6674024

[B346] Zhong G. Droho S. Crone S. A. Dietz S. Kwan A. C. Webb W. W. (2010). Electrophysiological characterization of V2a interneurons and their locomotor-related activity in the neonatal mouse spinal cord. *J. Neurosci.* 30 170–182. 10.1523/JNEUROSCI.4849-09.2010 20053899 PMC2824326

[B347] Ziskind-Conhaim L. Mentis G. Z. Wiesner E. P. Titus D. J. (2010). Synaptic integration of rhythmogenic neurons in the locomotor circuitry: the case of Hb9 interneurons. *Ann. N. Y. Acad. Sci.* 1198 72–84. 10.1111/j.1749-6632.2010.05533.x 20536922 PMC3057624

[B348] Zytnicki D. Lafleur J. Horcholle-Bossavit G. Lamy F. Jami L. (1990). Reduction of Ib autogenetic inhibition in motoneurons during contractions of an ankle extensor muscle in the cat. *J. Neurophysiol.* 64 1380–1389. 10.1152/jn.1990.64.5.1380 2283534

